# Differential Roles of Circular RNAs and Their Homologous Linear RNAs in *Hevea brasiliensis* Immunity Against *Erysiphe quercicola*

**DOI:** 10.3390/plants15071068

**Published:** 2026-03-31

**Authors:** Changshu Li, Lin Wang, Lijuan He, Xiao Li, Wenbo Liu, Chunhua Lin, Weiguo Miao

**Affiliations:** 1 Key Laboratory of Green Prevention and Control of Tropical Plant Diseases and Pest, Ministry of Education, School of Tropical Agriculture and Forestry, Hainan University, Haikou 570228, China; lichangshu1213@163.com (C.L.); helijuan741@163.com (L.H.); 993773@hainanu.edu.cn (X.L.); saucher@hainanu.edu.cn (W.L.); lin3286320@hainanu.edu.cn (C.L.); 2 Sanya Institute of Breeding and Multiplication, Sanya 572024, China; 3 Danzhou Invasive Species Observation and Research Station of Hainan Province, Hainan University, Danzhou 571737, China; 4 College of Life and Environmental Science, Wenzhou University, Wenzhou 325035, China; 5 School of Life and Health Science, Hainan University, Haikou 570228, China

**Keywords:** *Erysiphe quercicola*, circular RNAs, plant immunity, disease resistance, differential expression

## Abstract

*Hevea brasiliensis* (*H. brasiliensis*) is the principal source of natural rubber, but its productivity is severely threatened by powdery mildew caused by the biotrophic fungus *Erysiphe quercicola* (*E. quercicola*). Although circular RNAs (circRNAs) are emerging as key regulators in plant stress responses, their functions in *H. brasiliensis* immunity remain largely unexplored. Here, we aimed to systematically characterize circRNAs involved in the early immune response of *H. brasiliensis* to *E. quercicola*. Transcriptome sequencing and bioinformatic analyses identified 52 and 177 differentially expressed circRNAs (HbcircRNAs) at 1 and 3 days post-inoculation, respectively. Twelve HbcircRNAs with significant expression changes were validated, and nine were confirmed as true circRNAs. Functional assays using spray-induced gene silencing (SIGS) in *H. brasiliensis* leaves and heterologous overexpression in the *Arabidopsis thaliana eds1* mutant, which is susceptible to *E. quercicola*, revealed that HbcircARF3, HbcircSCSA1, and HbcircARFGAP8, together with their homologous linear counterparts (HbLinearRNAs), exert distinct regulatory effects on disease resistance. Silencing of these HbcircRNAs enhanced host immunity, whereas overexpression increased susceptibility. Pathway analyses suggested their involvement in auxin signaling, mitochondrial energy metabolism and vesicle trafficking. Collectively, our findings uncover the differential regulatory roles of circular and linear RNAs in the *H. brasiliensis–E. quercicola* interaction, providing mechanistic insights and potential molecular targets for breeding disease-resistant *H. brasiliensis*.

## 1. Introduction

*Hevea brasiliensis* (*H. brasiliensis*), the core source of natural rubber, is widely cultivated in tropical and subtropical regions of Asia and South America. The natural rubber produced by this species possesses irreplaceable strategic value in the fields of medical devices, transportation, and industrial production [1]. However, its productivity is severely threatened by powdery mildew caused by the biotrophic fungus *E. quercicola* [2]. As an obligate biotrophic pathogen [3,4], *E. quercicola* primarily infects young tissues such as buds and tender leaves of *H. brasiliensis* [5]. In severe cases, it induces leaf curling, chlorosis, and abscission, thereby impairing tree growth and reducing latex yield [6]. Although preliminary studies have examined the pathogenic mechanisms of powdery mildew in *H. brasiliensis*, the molecular responses and regulatory networks during the *H. brasiliensis*–*E. quercicola* interaction, particularly at early infection stages, remain largely unclear. This knowledge gap limits our understanding of disease resistance mechanisms and hinders the development of early prevention strategies and sustainable control methods against *H. brasiliensis* powdery mildew.

Circular RNAs (circRNAs) are a class of covalently closed single-stranded RNA molecules formed through noncanonical or back-splicing events [7]. They are widely distributed across both prokaryotes and eukaryotes [8,9]. CircRNAs exhibit diverse biological functions: they can act as microRNA (miRNA) sponges to regulate gene expression via the competing endogenous RNA (ceRNA) mechanism [10], and some even possess protein-coding potential [11,12,13]. Exon-derived circRNAs originate from protein-coding genes and share high sequence similarity with their linear homologs, enabling them to participate in post-transcriptional regulatory processes through synergistic or competitive interactions with parental genes [14]. In plants, circRNAs often display significant differential expression patterns under hormone signaling and stress conditions [15,16], indicating their involvement in regulating plant growth, development, and intercellular communication [17].

Increasing evidence suggests that circRNAs play critical roles in plant responses to both abiotic and biotic stresses. For example, in rice, Ye et al. identified 27 exon-derived circRNAs that were differentially expressed under phosphate-sufficient and phosphate-deficient conditions, implying their involvement in phosphate homeostasis and stress responses [18]. In *Arabidopsis thaliana*, overexpression of circGORK-derived from the outward-rectifying potassium channel gene conferred hypersensitivity to abscisic acid during germination and enhanced drought tolerance, providing direct evidence of circRNA-mediated regulation in abiotic stress [19]. In biotic stress contexts, 199 differentially expressed circRNAs were identified between insect-resistant and -susceptible soybean genotypes subjected to cotton bollworm herbivory [20]. Similarly, 32 and 83 specifically expressed circRNAs were detected in virus-infected and healthy tomato leaves, respectively, in plants infected with tomato yellow leaf curl virus (TYLCV), suggesting a global downregulation of circRNAs upon viral infection [21]. Furthermore, transgenic rice plants overexpressing circR5g05160 exhibited markedly enhanced resistance to *Magnaporthe oryzae* [22]. Collectively, these findings highlight that circRNAs exert diverse and significant regulatory functions in plant adaptation to environmental stimuli and pathogen attack.

Despite these advances, research on circRNAs in perennial woody species, particularly their roles in fungal defense, remains limited. In *H. brasiliensis*, previous studies have focused mainly on circRNAs in latex-producing tissues [23], while the functions of circRNAs in leaf immunity are still poorly characterized. Although circRNAs have been shown to exhibit significant differential expression in response to various stresses [15,16] and to play essential regulatory roles in plant immunity [24,25], their contributions to the early defense responses of *H. brasiliensis* against *E. quercicola* remain unexplored. *Hevea brasiliensis* leaves are divided into four developmental stages: bronze stage, color-changing stage, pale-green stage, and mature stage. The bronze stage refers to the young, newly expanded *Hevea brasiliensis* leaves that exhibit a characteristic bronze color. At this stage, leaves are tender and highly susceptible to powdery mildew infection, which is why this developmental stage was selected for inoculation and analysis. In this study, we established an interaction system between bronze-stage *H. brasiliensis* leaves and *E. quercicola* to investigate circRNA-mediated immune regulation. Using high-throughput RNA sequencing and bioinformatic analyses, we identified a set of differentially expressed circRNAs (HbcircRNAs) responsive to *E. quercicola* infection. The authenticity of selected HbcircRNAs was experimentally verified through convergent and divergent primer PCR, Ribonuclease R (RNase R) digestion, and Sanger sequencing. Furthermore, we explored their potential functions in immune regulation using SIGS in *H. brasiliensis* leaves and heterologous overexpression in the *Arabidopsis thaliana eds1* mutant, which is susceptible to *E. quercicola* [26].

This study reveals that key HbcircRNAs—such as HbcircARF3, HbcircSCSA1, and HbcircARFGAP8—and their homologous linear transcripts exert distinct regulatory effects on plant immunity. Our findings elucidate the potential molecular mechanisms by which circRNAs participate in the early defense response of *H. brasiliensis* against *E. quercicola*, providing new insights into the noncoding RNA-mediated immune network and offering theoretical guidance for molecular breeding and environmentally friendly disease management of *H. brasiliensis* diseases.

## 2. Results

### 2.1. Establishment of HbcircRNA Database and Bioinformatics Analysis

To investigate the expression dynamics of circular RNAs (HbcircRNAs) in bronze-stage leaves of *H. brasiliensis* during the early phase of *E. quercicola* infection, fungal colonization was first established and confirmed. Successful infection was verified by both phenotypic observation and molecular detection. The amount of pathogen-specific DNA at 3 days post-inoculation (dpi) was significantly higher than that at 1 dpi (*p* < 0.01), whereas no signal was detected in the mock-inoculated control (Supplementary Figure S1). These results confirmed the effective colonization of *E. quercicola* and provided a reliable foundation for subsequent transcriptomic analysis.

**Figure 1:**
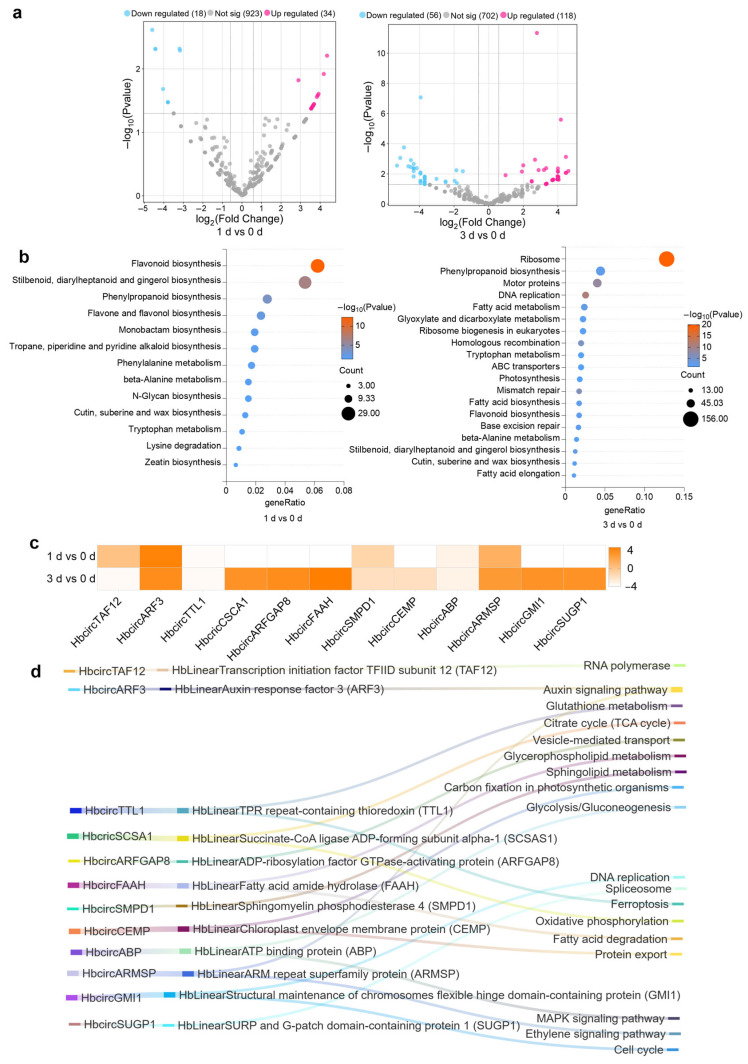
Differential expression, type distribution, functional enrichment, and pathway association with homologous linear RNAs of HbcircRNAs in bronze-stage *H. brasiliensis* leaves during early *E. quercicola* infection. (**a**) Volcano plot analysis of differentially expressed HbcircRNAs. The (**left**) panel corresponds to 1 d vs. 0 d, and the (**right**) panel corresponds to 3 d vs. 0 d. Each dot represents one HbcircRNA; dot colors indicate differential expression types (blue: down-regulated; gray: no significant difference; red: up-regulated). The *x*-axis denotes the logarithm of fold change (log_2_(Fold Change)), and the *y*-axis denotes the negative logarithm of the significance level (−log_10_(Pvalue)). (**b**) KEGG functional enrichment bubble plot of differentially expressed HbcircRNAs. The (**left**) sub-panel shows enrichment results for 1 d vs. 0 d, and the (**right**) sub-panel shows results for 3 d vs. 0 d. Bubble size represents the number of HbcircRNAs enriched in the pathway, and bubble color intensity corresponds to enrichment significance (−log_10_(Pvalue)). (**c**) Expression heatmap of 12 candidate HbcircRNAs. The heatmap displays the fold change of candidate HbcircRNAs at 1 dpi and 3 d relative to 0 d; the color gradient from blue to orange indicates expression changes from down-regulation to up-regulation (the corresponding value is log_2_(Fold Change)), which intuitively presents the expression dynamics of each candidate molecule across different infection time points. (**d**) Sankey diagram analysis of the association among HbcircRNAs, homologous HbLinearRNAs, and KEGG pathways. The (**left**) column lists 12 HbcircRNAs, the (**middle**) column lists their corresponding homologous HbLinearRNAs, and the (**right**) column lists associated KEGG pathways. Streamlines of different colors represent the correspondence between molecules and pathways, visually illustrating the functional pathway network involved in the HbcircRNA–HbLinearRNA module.

Using high-throughput RNA sequencing combined with DCC software-based analysis, the dynamic expression profiles of HbcircRNAs at different infection stages were systematically characterized. Differentially expressed HbcircRNAs (DEcircRNAs) were identified using the criteria |log_2_(Fold Change)| ≥ 1.5 and *p* ≤ 0.05. Volcano plot (Figure 1a) analysis revealed that, relative to 0 d, 52 DEcircRNAs were detected at 1 d (34 up-regulated and 18 down-regulated), whereas 177 DEcircRNAs were identified at 3 d (118 up-regulated and 59 down-regulated). The number of up-regulated HbcircRNAs was consistently approximately twice that of down-regulated ones, suggesting that *H. brasiliensis* may activate HbcircRNA expression as part of its early immune response to *E. quercicola* infection. Given that exonic regions usually encode proteins with critical physiological functions, the circRNAs identified in this study are predominantly exon-derived, and we investigated their roles in the plant defense response of *Hevea brasiliensis* under *Erysiphe quercicola* stress.

Functional annotation of DEcircRNAs using KEGG pathway enrichment revealed distinct stage-specific patterns (Figure 1b). At 1 d, DEcircRNAs were primarily enriched in secondary metabolic pathways such as flavonoid and phenylpropanoid biosynthesis, both of which are closely associated with the synthesis of early defense-related compounds. This suggests that certain HbcircRNAs may participate in rapid defense activation during the initial infection stage. By contrast, at 3 d, DEcircRNAs were significantly enriched in pathways related to ribosome biogenesis, DNA replication, and carbon fixation in photosynthetic organisms, indicating potential involvement in immune regulation and energy metabolism during later infection.

To identify key regulators, 12 candidate HbcircRNAs with significant expression changes were selected for further analysis, including HbcircTAF12 (*Transcription initiation factor TFIID subunit 12*), HbcircARF3 (*Auxin response factor 3*), HbcircTTL1 (*TPR repeat-containing thioredoxin*), HbcircSCSA1 (*Succinate-CoA ligase ADP-forming subunit α-1*), HbcircARFGAP8 (*ADP-ribosylation factor GTPase-activating protein 8*), HbcircFAAH (*Fatty acid amide hydrolase*), HbcircSMPD1 (*Sphingomyelin phosphodiesterase 4*), HbcircCEMP (*Chloroplast envelope membrane protein*), HbcircABP (*ATP-binding protein*), HbcircARMSP (*ARM repeat superfamily protein*), HbcircGMI1 (*Structural maintenance of chromosomes flexible hinge domain-containing protein*), and HbcircSUGP1 (*SURP and G-patch domain-containing protein 1*).

The expression heatmap (Figure 1c) displayed distinct temporal expression profiles of these 12 HbcircRNAs. Among them, HbcircTAF12, HbcircTTL1, HbcircSMPD1, HbcircCEMP, and HbcircABP were significantly down-regulated at both 1 d and 3 d, implying potential negative regulatory roles during infection. In contrast, HbcircARF3 exhibited continuous up-regulation across both time points, suggesting its involvement in sustained defense signaling. Several others, including HbcircSCSA1, HbcircARFGAP8, and HbcircSUGP1, were transiently down-regulated at 1 d and up-regulated at 3 d, indicating possible participation in immune activation during pathogen colonization.

A Sankey diagram (Figure 1d) illustrated the associations among the 12 HbcircRNAs, their homologous linear transcripts (HbLinearRNAs), and enriched KEGG pathways, highlighting key processes such as signal transduction, energy metabolism, and protein synthesis. Taken together, these results demonstrate that HbcircRNAs play vital and diverse roles in the early defense response of *H. brasiliensis* against *E. quercicola*. In particular, exon-derived HbcircRNAs appear to contribute significantly to the initial immune response, providing essential candidates for subsequent functional studies aimed at elucidating their regulatory mechanisms.

### 2.2. Validation of HbcircRNAs

To verify the 12 candidate HbcircRNAs identified from transcriptome analysis, convergent and divergent primers were designed based on their predicted back-splice junctions. Each candidate was examined using RNase R digestion and Sanger sequencing to assess the accuracy of the computational predictions. For each circRNA, one pair of convergent primers and one pair of divergent primers were designed. Convergent primers were used to amplify linear transcripts as controls, whereas divergent primers were used to detect the junction sites specific to circular RNAs. Three templates—RNase R-treated cDNA, untreated cDNA, and genomic DNA (gDNA)—were used in parallel PCR reactions. The comparison of amplification results from different primer–template combinations was used to distinguish circular molecules from linear ones.

**Figure 2:**
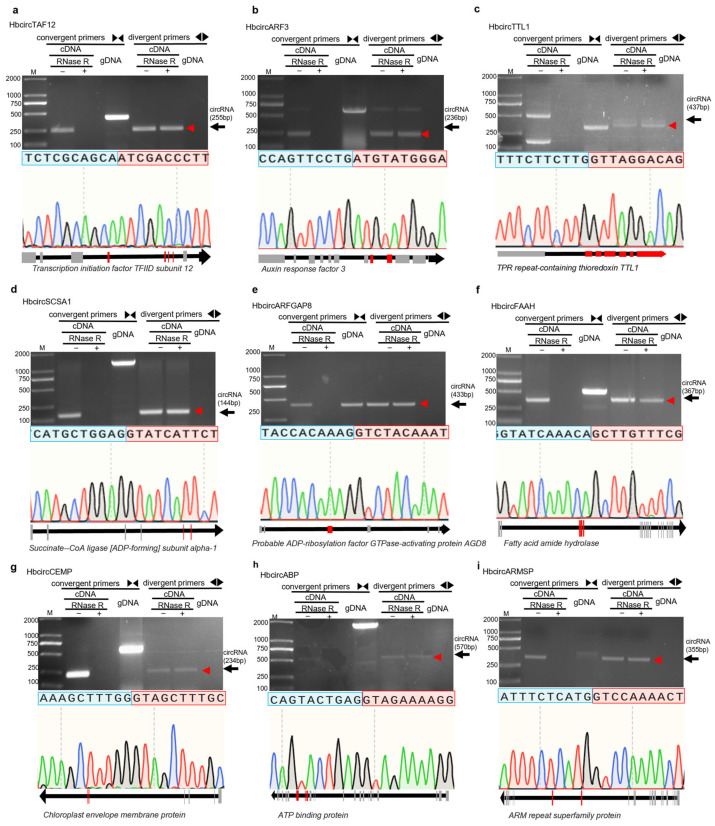
Validation of the circular structure authenticity of HbcircRNAs via convergent/divergent primer PCR and Sanger sequencing. (**a**–**i**) The validation results of 9 candidate HbcircRNAs (HbcircTAF12, HbcircARF3, HbcircTTL1, HbcircSCSA1, HbcircARFGAP8, HbcircFAAH, HbcircCEMP, HbcircABP, HbcircARMSP), respectively. The sequence above each panel represents the sequencing result of the back-splice junction (BSJ) region of the corresponding HbcircRNA, where the back-splice junction site is marked by a red box and a blue box. The chromatogram corresponds to the sequencing result of the BSJ region. Below each chromatogram, the name of the homologous parent gene corresponding to the HbcircRNA, as well as the exon positions from which the circRNA is derived, are labeled. The target bands are indicated by red arrows.

Divergent primers amplified clear bands only from RNase R-treated cDNA, while no products were observed in reactions using gDNA as the template. In contrast, convergent primers produced bands from both untreated cDNA and gDNA. Among the 12 candidates, nine, HbcircTAF12, HbcircARF3, HbcircTTL1, HbcircSCSA1, HbcircARFGAP8, HbcircFAAH, HbcircCEMP, HbcircABP, and HbcircARMSP, showed the expected amplification pattern for circular RNAs (Figure 2). These nine RNAs also remained detectable after RNase R treatment, indicating that they possess circular structures with higher stability than their linear counterparts. The remaining three candidates were not detected, which may be related to their low expression levels or inaccurate back-splice predictions in the bioinformatic analysis.

PCR products obtained with divergent primers were further analyzed by Sanger sequencing. The sequencing results showed distinct back-splice junctions in all nine verified HbcircRNAs, and the chromatograms were consistent with the corresponding genomic alignments (Figure 2). The exon composition and splicing directions were determined for each circRNA. HbcircTAF12 was derived from exons 4–7 of the *Transcription initiation factor TFIID subunit 12* gene; HbcircARF3 from exons 8–9 of the *Auxin response factor 3* gene; HbcircTTL1 from exons 2–7 of the *TPR repeat-containing thioredoxin* gene; HbcircSCSA1 from exons 5–6 of the *Succinate-CoA ligase ADP-forming subunit α-1* gene; HbcircARFGAP8 from exon 3 of the *ADP-ribosylation factor GTPase-activating protein* gene; HbcircFAAH from exons 3–5 of the *Fatty acid amide hydrolase* gene; HbcircCEMP from exons 5–6 of the *Chloroplast envelope membrane protein* gene; HbcircABP from exons 16–18 of the *ATP-binding protein* gene; and HbcircARMSP from exons 15–16 of the *ARM repeat superfamily protein* gene.

These results confirm that nine of the twelve candidates identified by transcriptome analysis exhibit circular structures verified by PCR and sequencing. The observed RNase R resistance and back-splice junctions support the presence of these circRNAs in *H. brasiliensis* leaves during *E. quercicola* infection. The remaining three candidates were not validated under the current experimental conditions. The validated circRNAs will be used in subsequent analyses to explore their potential roles in the interaction between *H. brasiliensis* and *E. quercicola*.

### 2.3. Effects of HbcircRNAs and Homologous Homologous HbLinearRNA Silencing on H. brasiliensis Resistance to Powdery Mildew

To investigate whether HbcircRNAs are involved in the interaction between *H. brasiliensis* and powdery mildew, six HbcircRNAs that were up-regulated during the early stage of *E. quercicola* infection, HbcircARF3, HbcircSCSA1, HbcircARFGAP8, HbcircFAAH, HbcircCEMP, and HbcircARMSP, were selected for further experiments. The corresponding genes were targeted and silenced in *H. brasiliensis* leaves using SIGS technology, and the resulting disease resistance phenotypes were evaluated. Based on the structural and functional characteristics of HbcircRNAs, double-stranded RNA (dsRNA) products were synthesized through in vitro transcription using a T7 promoter. Templates used for transcription consisted of 21 bp sequences spanning the back-splice junctions of each candidate HbcircRNA. Meanwhile, double-stranded RNA of green fluorescent protein (GFP-dsRNA) was synthesized using the same in vitro transcription method with the GFP coding sequence as a template and served as the experimental control. Similarly, the corresponding HbLinearRNA-dsRNA products were synthesized in vitro using the same approach, and the disease resistance of *H. brasiliensis* leaves was analyzed after silencing their homologous linear transcripts.

Quantitative reverse transcription PCR (qRT-PCR) was performed to determine the relative expression levels of each target gene in both HbcircRNA-dsRNA and HbLinearRNA-dsRNA treatment groups at 0, 1, 3, 5, and 7 days post-inoculation (dpi) with *E. quercicola* (Supplementary Figure S2). The results showed that silencing one type of RNA affected the expression of the other in both HbcircRNA-dsRNA and HbLinearRNA-dsRNA treatments, suggesting possible regulatory interactions between the two transcript forms. For example, in the HbcircFAAH-dsRNA and HbcircARMSP-dsRNA treatment groups, the expression levels of the corresponding HbLinearRNAs were significantly increased after silencing their respective HbcircRNAs, suggesting that HbcircFAAH and HbcircARMSP may negatively influence the expression of their homologous HbLinearRNAs.

**Figure 3:**
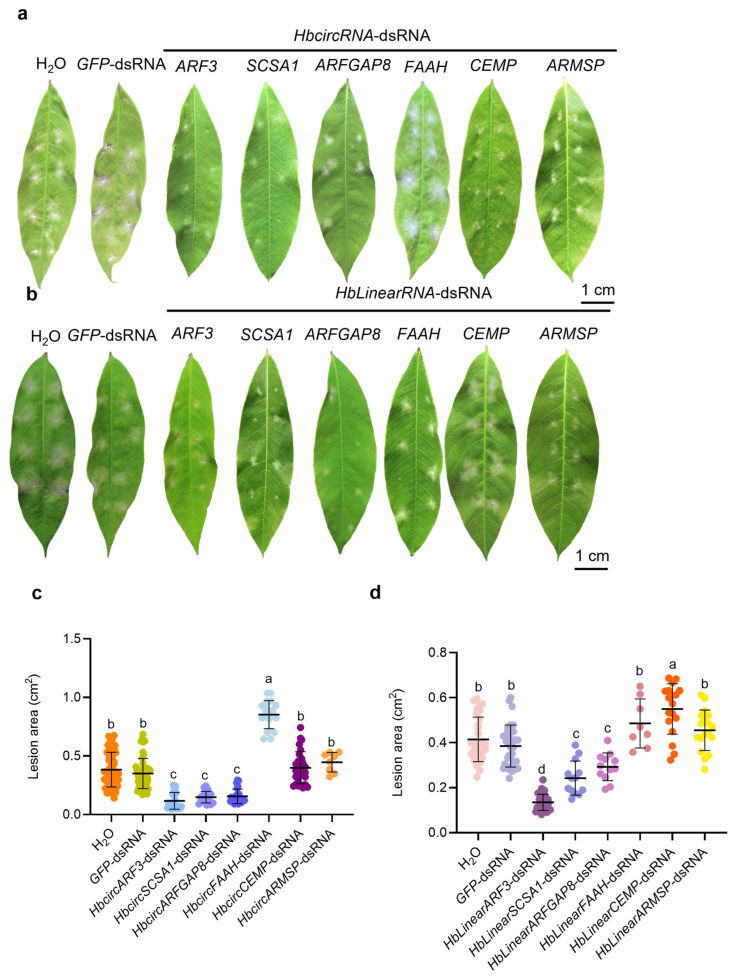
Silencing of HbcircRNAs and Their Homologous HbLinearRNAs Is Involved in Resistance to Powdery Mildew Growth in *H. brasiliensis* Leaves. (**a**,**b**) Phenotypes of powdery mildew lesions on *H. brasiliensis* leaves under silencing of HbcircRNAs and their homologous HbLinearRNAs at 7 days post inoculation (dpi). *H. brasiliensis* leaves were sprayed with a conidial suspension (1 × 10^5^ conidia/mL). (**c**,**d**) The percentage of lesion area per leaf under silencing of HbcircRNAs and their homologous HbLinearRNAs was calculated and statistically analyzed. Scatter plots show the mean values and standard deviations. Nine leaves from three independent experiments were examined. The mock was compared with each of the other samples using a two-tailed Student’s *t*-test. Significant differences are indicated by different letters.

At 7 dpi, disease phenotypes were assessed by measuring the lesion area on the leaf surface. Compared with both the blank control (Mock) and the negative control (GFP-dsRNA), treatment with HbcircARF3-dsRNA, HbcircSCSA1-dsRNA, and HbcircARFGAP8-dsRNA reduced the lesion area by 58.98%, 64.08%, and 73.39%, respectively (Figure 3a,c). In contrast, treatment with HbcircFAAH-dsRNA increased the lesion area by 86.03%, while HbcircCEMP-dsRNA and HbcircARMSP-dsRNA treatments showed no significant differences compared with the control groups. Similarly, in the HbLinearRNA-dsRNA treatment groups, the lesion area was reduced by 62.98%, 35.79%, and 26.05% in HbLinearARF3-dsRNA, HbLinearSCSA1-dsRNA, and HbLinearARFGAP8-dsRNA plants, respectively, relative to the control (Figure 3b,d). In contrast, treatment with HbLinearCEMP-dsRNA increased the lesion area by 39.21%, while HbLinearARMSP-dsRNA and HbLinearFAAH-dsRNA treatments produced no significant difference compared with the controls.

Together, these results indicate that HbcircRNAs and their homologous HbLinearRNAs respond to *E. quercicola* infection and that silencing either transcript type can influence the disease resistance phenotype of *H. brasiliensis* leaves. The changes in expression and lesion area measurements suggest that regulatory associations may exist between specific circRNAs and their corresponding linear transcripts, while the extent of such interactions and their biological consequences may vary among different gene pairs.

### 2.4. Immune Responses of H. brasiliensis Leaves to E. quercicola Infection Mediated by HbcircRNAs and Their Homologous HbLinearRNA Silencing

In plant–fungus interactions, the accumulation of reactive oxygen species (ROS) and callose is a common indicator of pattern-triggered immunity (PTI). The 2 dpi point represents a key stage at which *Erysiphe quercicola* has just finished penetration and begun haustorium formation. This is also when plants launch their strongest early defense responses, including callose deposition. To examine the possible roles of HbcircRNAs and their homologous HbLinearRNAs in the immune response of *H. brasiliensis* to *E. quercicola*, and to explore their potential functional relationships, spray-induced gene silencing (SIGS) was applied to target these RNAs separately. ROS accumulation and callose deposition were assessed at 2 dpi using DAB and aniline blue staining, respectively.

**Figure 4:**
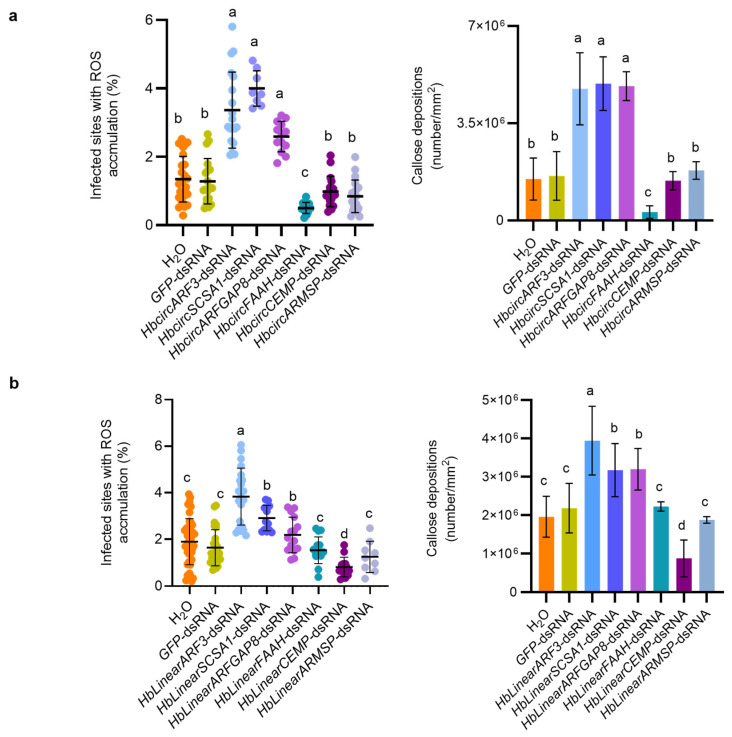
RNA Silencing Targeting HbcircRNAs and Their Homologous HbLinearRNAs Participates in ROS Accumulation and Callose Deposition During Powdery Mildew Infection in *H. brasiliensis* Leaves. (**a**) The percentage of infected sites with ROS accumulation and the amount of callose deposition after HbcircRNA silencing were calculated and statistically analyzed. Scatter plots and bar charts show the mean values and standard deviations, respectively. A total of 600 infection sites from three independent experiments were analyzed. The mock was compared with each of the other samples using a two-tailed Student’s *t*-test. Significant differences (*p* < 0.01) are indicated by letters. (**b**) The percentage of infected sites with ROS accumulation and the amount of callose deposition after HbLinearRNA silencing were calculated and statistically analyzed. Scatter plots and bar charts show the mean values and standard deviations, respectively. A total of 600 infection sites from three independent experiments were analyzed. The mock was compared with each of the other samples using a two-tailed Student’s *t*-test. Significant differences are indicated by different letters.

Phenotypic observation and quantitative analysis showed that silencing of HbcircARF3, HbcircSCSA1, and HbcircARFGAP8 resulted in higher ROS accumulation compared with the GFP-dsRNA control group, increasing by approximately 2.3-, 3.0-, and 2.0-fold, respectively (Figure 4a; Supplementary Figure S3). Correspondingly, callose deposition in these three treatments increased by 3.1-, 3.2-, and 3.1-fold, respectively. In contrast, silencing of HbcircFAAH led to a decrease in ROS accumulation by 62.1% and a reduction in callose deposition by 80.3% relative to the control. No significant differences in either ROS or callose accumulation were observed in the HbcircCEMP-dsRNA and HbcircARMSP-dsRNA groups compared with the control (Figure 4a).

The immune response patterns observed after silencing of homologous HbLinearRNAs were generally similar to those obtained from HbcircRNA silencing. In HbLinearARF3-, HbLinearSCSA1-, and HbLinearARFGAP8-dsRNA-treated plants, ROS accumulation increased by 2.1-, 1.6-, and 1.2-fold compared with the control, respectively, and callose deposition increased by 1.9-, 1.5-, and 1.5-fold (Figure 4b; Supplementary Figure S3). In the HbLinearCEMP-dsRNA treatment, ROS accumulation and callose deposition decreased by 45.3% and 38.0%, respectively, relative to the control. No clear differences in these indicators were detected between HbLinearFAAH-dsRNA or HbLinearARMSP-dsRNA plants and the control group (Figure 4b).

These observations suggest that both HbcircRNAs and their corresponding HbLinearRNAs are involved in the immune response of *H. brasiliensis* to *E. quercicola*, possibly through modulation of ROS production and callose deposition. Among them, HbcircARF3, HbcircSCSA1, HbcircARFGAP8, and their homologous HbLinearRNAs showed patterns consistent with negative regulation of immunity, as their silencing was associated with increased ROS and callose accumulation. In contrast, HbcircFAAH appeared to have an opposite effect, as its silencing reduced both immune-related indicators. HbcircCEMP, HbcircARMSP, and their corresponding HbLinearRNAs showed no measurable effect under the tested conditions.

### 2.5. Overexpression of HbcircRNAs and Their Homologous HbLinearRNA Genes Mediate the Interaction Between Arabidopsis thaliana and E. quercicola

Considering the limitations of transgenic manipulation in *H. brasiliensis*, the immune-related roles of HbcircRNAs and their homologous HbLinearRNAs in the interaction between *H. brasiliensis* and *E. quercicola* were further examined using a transgenic *Arabidopsis thaliana* overexpression system. Transgenic *Arabidopsis* plants were generated via *Agrobacterium*-mediated transformation. Because the vector carried a herbicide resistance gene, sterilized seeds were cultured and screened on MS medium supplemented with herbicide under sterile conditions.

To confirm the identity of the positive HbcircRNA-overexpression (HbcircRNA-OE) lines, a primer pair was designed with an upstream primer located within the 35S promoter region of the vector and a downstream primer within the target insert sequence. PCR amplification was carried out using genomic DNA extracted from herbicide-resistant plants as templates, and the genomic DNA of Δ*eds1 Arabidopsis* was used as the negative control. The presence of correctly sized amplification bands was confirmed by agarose gel electrophoresis, identifying the positive transgenic lines. For the verification of HbLinearRNA-overexpression (HbLinearRNA-OE) plants, transgene expression was further examined by Western blotting. In addition, because circRNA sequences are short and may potentially interfere with endogenous homologous genes in *Arabidopsis*, a circRNA-specific RNA interference (HbcircRNA-RNAi) vector was constructed to reduce cross-interference. These validations confirmed that the screened plants were positive transgenic lines (Supplementary Figure S4). The verified plants were then inoculated with *E. quercicola*, and the lesion area, reactive oxygen species (ROS) production, and callose deposition were analyzed.

**Figure 5:**
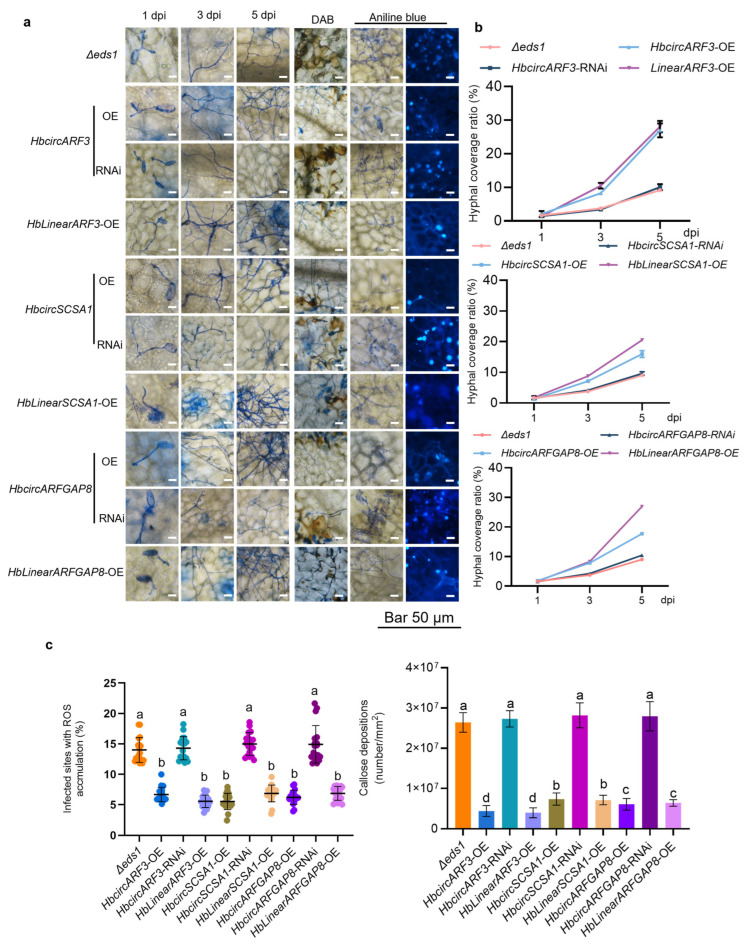
Expression of HbcircARF3/SCSA1/ARFGAP8 and Their Homologous HbLinearRNAs in *Arabidopsis thaliana* Participates in the Immune Interaction with *E. quercicola*. (**a**) Expression of HbcircARF3/SCSA1/ARFGAP8 and their homologous linear RNAs affects the growth of *E. quercicola* and triggers immune responses in *Arabidopsis thaliana* leaves. Microscopic observations were performed on hyphal coverage density at 1, 3, and 5 dpi (days post-inoculation), as well as reactive oxygen species (ROS) accumulation at infection sites (detected by DAB staining) and callose accumulation at infection sites (detected by aniline blue staining) in Δ*eds1* leaves challenged with *E. quercicola* at 2 dpi. Scale bar, 50 μm. (**b**) Expression of HbcircARF3/SCSA1/ARFGAP8 and their homologous linear RNAs affects the growth of *E. quercicola*. Statistical analysis of hyphal coverage density was conducted on Δ*eds1* leaves overexpressing HbcircRNAs and their homologous linear RNAs, or subjected to HbcircRNA-RNAi, at 1, 3, and 5 dpi (days post-inoculation). Scale bar, 50 μm. N = 6, data are presented as mean ± standard deviation (SD). (**c**) DAB and aniline blue staining reveal reactive oxygen species (ROS) and callose accumulation at the infection sites of Δ*eds1* leaves challenged with *E. quercicola*. Scale bar, 50 μm. The percentage of infected sites with ROS accumulation and the accumulation level of callose at infected sites were calculated and statistically analyzed. Scatter plots show the mean and standard deviation values. A total of 600 infection sites from three independent experiments were analyzed. The mock group was compared with each of the other samples using a two-tailed Student’s *t*-test. Significant differences (*p* < 0.01) are indicated by letters.

Aniline blue staining and hyphal growth analysis at 1, 3, and 5 days post-inoculation (dpi) showed that the hyphal expansion rate was higher in HbcircARF3-OE, HbcircSCSA1-OE, and HbcircARFGAP8-OE plants than in the Δ*eds1* control group, with denser hyphal branching (Figure 5a,b). The hyphal coverage in HbcircARF3-OE plants reached 1.6 times that in Δ*eds1* plants at 5 dpi, while that in HbLinearARF3-OE plants was approximately 2.1 times higher. Similarly, the hyphal expansion in HblnearSCSA1-OE and HbLinearARFGAP8-OE plants was greater than in the Δ*eds1* control group. No significant differences in hyphal growth were observed between HbcircRNA-RNAi plants and the Δ*eds1* control. These data indicate that both HbcircRNA and HbLinearRNA overexpression increased the susceptibility of *Arabidopsis* to *E. quercicola* infection, and that the effect of HbLinearRNA overexpression was consistently stronger.

DAB and aniline blue staining results at 2 dpi showed consistent trends (Figure 5a,c). ROS accumulation in HbcircARF3-OE, HbcircSCSA1-OE, and HbcircARFGAP8-OE plants was 58.52%, 64.98%, and 58.42% lower, respectively, than in the Δ*eds1* control. Callose deposition was also reduced by 83.99%, 73.14%, and 77.91%, respectively. In HbLinearRNA-OE plants, ROS levels were reduced by 59.13%, 50.14%, and 54.35%, respectively, compared with the control, while callose deposition decreased by 85.52%, 74.31%, and 76.69%, respectively. No significant differences were observed between HbcircRNA-RNAi plants and the Δ*eds1* control group.

Overall, these results show that overexpression of HbcircARF3, HbcircSCSA1, HbcircARFGAP8, and their homologous HbLinearRNAs was associated with enhanced hyphal growth and reduced immune-associated responses in *Arabidopsis* following *E. quercicola* inoculation. Under the tested conditions, the reduction in ROS production and callose accumulation, together with increased hyphal proliferation, suggest that both HbcircRNAs and HbLinearRNAs can modulate host susceptibility. The effect of HbLinearRNA overexpression was generally more pronounced than that of the corresponding HbcircRNAs. These results are consistent with the observations in *H. brasiliensis*, indicating that the regulation of immunity-related phenotypes by these homologous RNA pairs may be at least partially conserved, though differing in intensity. The cross-species analysis in *Arabidopsis* therefore provides supportive experimental evidence for the potential regulatory relationships between HbcircRNAs and HbLinearRNAs in plant–pathogen interactions.

### 2.6. Dynamic Expression of Downstream Pathway Genes Involved in the Immunity of H. brasiliensis Against E. quercicola Mediated by HbcircARF3, HbcircSCSA1 and HbcircARFGAP8

**Figure 6:**
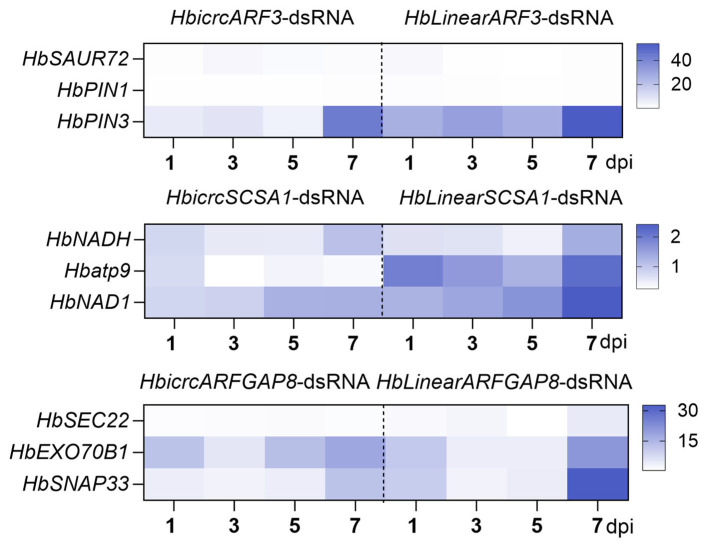
Heatmap of Expression Dynamics of Key Downstream Pathway Genes After HbcircARF3/SCSA1/ARFGAP8 and Their Homologous HbLinearRNA Treatments. This figure is an expression heatmap illustrating the relative expression levels of key genes across three core pathways at 1, 3, 5, and 7 dpi following *E. quercicola* infection, under two distinct dsRNA treatments: HbcircRNA-dsRNA (a silencing treatment targeting HbcircARF3, HbcircSCSA1, and HbcircARFGAP8) and HbLinearRNA-dsRNA (a silencing treatment targeting the homologous HbLinearRNAs corresponding to these HbcircRNAs). The heatmap is divided into three independent modules, each corresponding to one of the HbcircRNAs and its regulated pathway; for each module, the *x*-axis denotes the time points after treatment (in dpi), the *y*-axis lists the key genes of the relevant pathway, and the color bar positioned to the right of each module serves as a reference for relative expression levels—where the values on the color bar indicate the range of expression levels, and a darker color corresponds to a higher relative expression level of the gene.

To further explore the regulatory roles of *HbcircARF3*, *HbcircSCSA1*, *HbcircARFGAP8* and their homologous HbLinearRNAs on downstream genes in the immune response of *H. brasiliensis* against *E. quercicola*, the expression dynamics of key genes involved in disease-resistant pathways were detected via qRT-PCR. Leaf samples were collected at 1, 3, 5 and 7 dpi after treatment with HbcircRNA-dsRNA or HbLinearRNA-dsRNA and inoculation with *E. quercicola*, and the expression dynamics were visualized via heatmaps (Figure 6).

For HbcircARF3, the expression changes of key genes in the auxin signaling pathway, including *HbSAUR72* (*Small Auxin-Up RNA 72*), *HbPIN1* (*PIN-FORMED 1*) and *HbPIN3* (*PIN-FORMED 3*), were focused on. The heatmap showed that under HbcircARF3-dsRNA treatment, the relative expression level of the auxin transport gene *HbPIN3* was significantly upregulated at 7 dpi, whereas no significant fluctuations were observed in the expression of the auxin response gene *HbSAUR72* and another transport gene *HbPIN1* at all time points. Under HbLinearARF3-dsRNA treatment, *HbPIN3* was also upregulated at 7 dpi, suggesting that HbcircARF3 may affect the auxin distribution at pathogen infection sites by specifically regulating the expression of the auxin transport gene *HbPIN3*, thereby participating in the immune response.

For HbcircSCSA1, the expression dynamics of genes involved in the mitochondrial respiration pathway, including *HbNADH* (*Nicotinamide Adenine Dinucleotide*), *Hbatp9* (*ATP synthase subunit 9*) and *HbNAD1* (*NADH dehydrogenase subunit 1*), were detected. The results showed that after HbcircSCSA1-dsRNA treatment, the expression of the mitochondrial respiratory chain gene *HbNAD1* was continuously upregulated from 5 to 7 dpi, and *HbNADH* was moderately upregulated at 7 dpi. Under HbLinearSCSA1-dsRNA treatment, the ATP synthase gene *Hbatp9* was upregulated from 3 to 7 dpi, and *HbNAD1* was upregulated at 7 dpi. These findings indicate that HbcircSCSA1 mainly affects the immune metabolic process by regulating the expression of mitochondrial respiratory chain genes, whereas its homologous HbLinearRNAs tend to regulate energy metabolism-related genes such as ATP synthesis-related genes, with significant differences in downstream targets between the two.

Finally, for HbcircARFGAP8, the expression changes of key genes in the vesicle trafficking pathway, including *HbSEC22* (*Secretory Pathway 22*), *HbEXO70B1* (*Exocyst complex component 70B1*) and *HbSNAP33* (*soluble N-ethylmaleimide-sensitive factor adaptor protein 33*), were analyzed. The heatmap showed that after HbcircARFGAP8-dsRNA treatment, the expression of the vesicle fusion-related gene *HbSNAP33* was significantly upregulated at 7 dpi, and the vesicle trafficking gene *HbEXO70B1* was fluctuantly upregulated at 1, 5 and 7 dpi. Under HbLinearARFGAP8-dsRNA treatment, *HbSNAP33* was also upregulated at 7 dpi, suggesting that HbcircARFGAP8 may participate in the intercellular transport and secretion of immunity-related proteins by regulating the expression of vesicle trafficking-related genes.

These findings reveal the unique regulatory roles of HbcircRNAs in the immune response of *H. brasiliensis* against *E. quercicola*, providing novel pathway evidence for subsequent studies on the molecular mechanism underlying the interaction between *H. brasiliensis* and *E. quercicola*.

## 3. Discussion

In the present work, two complementary approaches—SIGS-based silencing in *Hevea brasiliensis* and ectopic overexpression in *Arabidopsis*—provided evidence that HbcircARF3, HbcircSCSA1, HbcircARFGAP8 and their homologous linear RNAs (HbLinearRNAs) may serve as negative immune regulators during plant resistance to *E. quercicola*. The circular structures of nine candidate molecules were experimentally verified, and their functional characteristics were examined using SIGS and *Arabidopsis thaliana* overexpression systems. The results indicated that HbcircARF3, HbcircSCSA1, HbcircARFGAP8, and their homologous HbLinearRNAs may function as negative regulators of immunity against *E. quercicola*, influencing the disease-resistance phenotype of *H. brasiliensis* leaves. In addition, the regulatory effects of HbLinearRNAs appeared stronger than those of their homologous circular forms. Analysis of downstream pathways further showed that these HbcircRNAs affect disease resistance by modulating key genes involved in auxin signaling, mitochondrial respiration, and vesicle trafficking.

### 3.1. Conservation of Functional Relationships Between HbcircRNAs and HbLinearRNAs

In this study, two experimental systems—SIGS-mediated silencing in *H. brasiliensis* and overexpression in *Arabidopsis*—demonstrated that HbcircARF3, HbcircSCSA1, HbcircARFGAP8, and their homologous HbLinearRNAs may all act as negative immune regulators involved in plant responses against *E. quercicola*. The regulatory activity of homologous HbLinearRNAs was significantly stronger than that of HbcircRNAs. This core finding is consistent with existing conclusions in the field of plant circRNA research, further supporting the functional conservation of the HbcircRNA-HbLinearRNA regulatory module.

Previous studies have shown that exon-derived circRNAs and their homologous linear RNAs can synergistically regulate plant defense responses against pathogens. For example, research on *Arabidopsis* resistance to bacterial and fungal pathogens has confirmed that exon-derived circRNAs and their homologous linear RNAs interact to form functionally complementary regulatory units [27]. Moreover, studies in maize and tomato have revealed that circRNAs and linear RNAs exhibit distinct regulatory intensities in immune regulation, which aligns closely with the results of this study [28,29]. Collectively, these findings highlight the conserved characteristics of non-coding RNAs in regulating plant immune responses. Combining evidence from multiple crops such as *Arabidopsis*, maize, and tomato, it can be inferred that in *H. brasiliensis*, a dicotyledonous economic crop, the circRNA–linear RNA regulatory module reflects an evolutionarily conserved mechanism shared by both monocotyledonous and dicotyledonous species.

### 3.2. Regulation of Disease Resistance Pathways by HbcircRNAs and Their Homologous HbLinearRNAs

Analysis of downstream pathway gene expression indicated that HbcircARF3 regulates the auxin transport gene HbPIN3, HbcircSCSA1 targets the mitochondrial respiratory chain gene HbNAD1, and HbcircARFGAP8 affects the vesicle trafficking gene HbSNAP33, whereas homologous HbLinearRNAs tend to regulate energy metabolism components such as ATP synthesis-related genes.

The auxin signaling pathway exerts dual regulatory functions in plant disease resistance: it not only participates in plant growth and development but also modulates immune responses by altering hormone distribution at pathogen infection sites [30,31,32]. As a potential regulatory target of HbcircARF3, the upregulation of HbPIN3 after HbcircARF3 silencing may promote the activation of plant defense-related genes by modifying auxin distribution at infection sites. The mitochondrial respiration pathway provides essential energy for immune metabolism [33,34]. Under HbcircSCSA1-dsRNA and HbLinearSCSA1-dsRNA treatments, the expression of HbNAD1 showed time-dependent upregulation, peaking at 7 dpi. As a core source of energy for immune metabolism [35], this upregulation reflects an enhanced respiration rate in the later stage of pathogen colonization, which may supply ATP for energy-demanding immune processes such as ROS production and defense protein synthesis [36,37].

Vesicle trafficking plays an important role in the intercellular transport and secretion of immunity-related proteins [38,39]. Under HbcircARFGAP8-dsRNA treatment, both the vesicle fusion gene HbSNAP33 and the trafficking gene HbEXO70B1 were upregulated, whereas HbLinearARFGAP8-dsRNA treatment caused only mild upregulation of HbSNAP33. This difference suggests that HbcircARFGAP8 may promote the intercellular transport and secretion of immune-related proteins by coordinating vesicle trafficking and fusion processes [40,41,42]. These pathways represent core modules of plant immune responses, and their target differentiation likely contributes to the variation in regulatory intensity between HbcircRNAs and HbLinearRNAs. This finding reveals the functional differentiation and potential synergy between HbcircRNAs and HbLinearRNAs in *H. brasiliensis* immunity against *E. quercicola*, offering precise targets and theoretical support for molecular breeding of disease-resistant cultivars.

### 3.3. Early Interaction Between H. brasiliensis and E. quercicola: Conserved and Species-Specific Features

The molecular characteristics of the early interaction between *H. brasiliensis* and *E. quercicola* observed in this study are consistent with general patterns of early plant–pathogen interactions. In the early stage of pathogen invasion, plants rapidly activate defense responses, including secondary metabolic pathways, accumulation of defense compounds, and pattern-triggered immunity (PTI), to restrict pathogen colonization. These responses are the basis of basal immunity [43]. The core of this immune mode lies in the recognition of pathogen-associated molecular patterns (MAMPs) by pattern recognition receptors (PRRs), which trigger downstream defense signaling cascades [44]. This mechanism is conserved across diverse plant–pathogen systems [32].

In the present study, at 1 dpi after *E. quercicola* inoculation, differentially expressed HbcircRNAs were mainly enriched in secondary metabolic pathways such as flavonoid and phenylpropanoid biosynthesis [45]. This finding is consistent with observations in other plants, suggesting that reinforcement of cell wall structure through secondary metabolites in the early stage is a common defense strategy against fungal invasion [46].

Moreover, the dynamic changes in ROS and callose accumulation observed in this study are consistent with the typical early activation features of PTI responses [47,48,49,50]. After pathogen infection, plants rapidly accumulate ROS, which can directly inhibit pathogens and serve as signaling molecules to activate downstream defense gene expression. Callose deposition reinforces cell walls to form a physical barrier that restricts pathogen spread [51]. These characteristics align with the marked ROS and callose accumulation detected in *H. brasiliensis* at 2 dpi after *E. quercicola* inoculation. ROS serves as a core node in early immune signaling networks, and its intensity and duration directly affect the efficiency of defense activation [52]. The localized deposition of callose at infection sites is a conserved defense mechanism observed in several species, including *Arabidopsis* and rice [53].

At 3 dpi, the enrichment of differentially expressed HbcircRNAs shifted from secondary metabolism to pathways such as ribosome biogenesis, DNA replication, and carbon fixation. This shift may represent an adaptive strategy by *H. brasiliensis* to balance defense responses with growth and development following pathogen colonization, reflecting species-specific characteristics in the *H. brasiliensis–E. quercicola* interaction. Studies on similar regulatory mechanisms in other plant species suggest that plants coordinate immune activation and growth costs through species-specific signaling regulation in early immune responses [54], providing a theoretical basis for the observed pathway transition in *H. brasiliensis*.

In conclusion, this study reveals the important roles of HbcircRNAs and their homologous HbLinearRNAs in the immune response of *H. brasiliensis* to *E. quercicola*. By regulating genes associated with auxin signaling, mitochondrial respiration, and vesicle trafficking pathways, these molecules jointly influence *E. quercicola* resistance. Moreover, the observed coordination between immune regulation and growth responses in *H. brasiliensis* provides a theoretical framework for molecular breeding and sustainable management of disease-resistant *H. brasiliensis*.

## 4. Materials and Methods

### 4.1. Plant Growth Conditions and Culture of E. quercicola

Grafted seedlings of *H. brasiliensis* cultivar Reyan 73397 were purchased from Danzhou, Hainan Province. They were cultured at 23 ± 1 °C with a relative humidity of 70% under a photoperiod of 16 h light/8 h dark. *Erysiphe quercicola* strain HO-73 was maintained on bronze-stage leaves of healthy *Hevea brasiliensis* seedlings (cultivar Reyan 73397) under greenhouse conditions (23 ± 1 °C, 70% relative humidity, 16 h light/8 h dark). Fresh conidia were obtained from 12-day-old infected leaves and used for inoculation experiments. Seeds of the *Arabidopsis thaliana* mutant Δ*eds1* (CS85829/N85829, Col-0 background) and *Nicotiana benthamiana* (NCBI Taxonomy ID: 4100, GRIN: PI 555684) were subjected to low-temperature stratification at 4 °C for 48 h, then sown in moist vermiculite and cultured at 22 ± 1 °C for 5–7 days. After germination, the seedlings were transplanted and grown in a greenhouse under the conditions of 22 ± 1 °C and a 16 h light/8 h dark photoperiod.

### 4.2. Extraction of Total RNA and Genomic DNA

Fresh conidia of *Erysiphe quercicola* strain HO 73 were used to inoculate bronze stage *Hevea brasiliensis* leaves. Mock inoculated plants treated with sterile water were used as controls. To avoid circadian rhythm effects, all samples were collected at the same time each day (between 9:00 and 10:00 a.m.). Inoculated and mock treated leaves were harvested at 0, 1, 3, 5 and 7 days post inoculation (dpi), which represent key early infection stages including conidial germination, appressorium formation, and penetration with haustorium establishment. Immediately after collection, all samples were snap frozen in liquid nitrogen and stored at −80 °C until RNA extraction. Total RNA was extracted from *H. brasiliensis* leaves using the CTAB method [55]. The ground samples were added to 10 mL of CTAB extraction buffer containing 2% CTAB, 1 mol/L Tris-HCl (pH = 8.0), 0.5 mol/L EDTA, 1.4 mol/L NaCl and 2% β-mercaptoethanol. The mixture was inverted to mix thoroughly and incubated in a water bath at 65 °C for 10 min. An equal volume (10 mL) of chloroform/isoamyl alcohol (24:1) was added, and the mixture was shaken well, followed by centrifugation at 11,429 rpm for 10 min at 4 °C. The supernatant was transferred to a new 50 mL centrifuge tube, and a 1/3 volume of 8 mol/L LiCl solution was added. The mixture was gently inverted to mix and then placed at −20 °C overnight. After centrifugation at 11,429 rpm for 20 min at 4 °C, the supernatant was discarded, and the precipitate was dissolved in 1 mL of Trizol Buffer. Then 0.2 mL of chloroform was added, and the mixture was centrifuged at 11,429 rpm for 15 min at 4 °C. The upper aqueous phase was transferred to a 1.5 mL centrifuge tube, mixed with 0.5 mL of isopropanol, and incubated at −20 °C for 30 min. The mixture was centrifuged at 11,429 rpm for 20 min at 4 °C, and the precipitate was washed with 1 mL of 70% ethanol prepared with DEPC-treated water, followed by centrifugation at 11,429 rpm for 10 min at 4 °C. The supernatant was discarded, and 1 mL of 75% ethanol was added. The mixture was shaken well and centrifuged at 11,429 rpm for 10 min at 4 °C. The precipitate was air-dried at room temperature and dissolved in 30 μL of DEPC-treated water, and the RNA concentration was determined before storage at −80 °C.

Genomic DNA was extracted from *H. brasiliensis* leaves using the CTAB method [56]. The ground samples were transferred to 1.5 mL centrifuge tubes, and 600 μL of preheated CTAB mixture (2× CTAB solution with 2% β-mercaptoethanol, preheated at 65 °C) was added. The mixture was vortexed for 20 s, incubated in a water bath at 65 °C for 40 min, and then placed on ice for 10 min. An equal volume of chloroform/isoamyl alcohol (24:1) was added, and the mixture was vortexed well, placed on ice for 20 min, and centrifuged at 11,895 rpm for 10 min at 4 °C. The supernatant was transferred to a new 1.5 mL centrifuge tube, mixed with 500 μL of pre-cooled isopropanol (4 °C), vortexed well, and placed on ice for 30 min. After centrifugation at 12,000 rpm for 10 min at 4 °C, 150 μL of 3 mol/L NaAc and 600 μL of anhydrous ethanol were added to the precipitate. The mixture was gently inverted to mix and placed on ice for 20 min. The mixture was centrifuged at 12,000 rpm for 20 min at 4 °C, and the precipitate was retained. The precipitate was washed twice with 70% ethanol, air-dried naturally, and dissolved in 100 μL of ddH_2_O. The DNA was stored at −20 °C for short-term use and at −80 °C for long-term storage.

### 4.3. Plasmid Construction

First-strand cDNA was synthesized from *Hevea brasiliensis* total RNA using HiScript III All-in-one RT SuperMix Perfect for qPCR (Vazyme, Nanjing, China) according to the manufacturer’s instructions. According to the kit manufacturer’s instructions: RNA: 1 pg–1 μg; 5× All-in-one qRT Super Mix: 4 μL; Enzyme Mix: 1 μL; RNase-free ddH_2_O: added to a final volume of 20 μL. After preparation of the reaction mixture, the reaction was incubated at 37 °C for 10–30 min, followed by inactivation at 70 °C for 10 min. To verify the authenticity and sequence accuracy of HbcircRNAs, the target fragments of HbcircRNAs were first cloned from *H. brasiliensis* cDNA and recombined into the pENTR vector via the restriction endonuclease Xcm I site. The primers used in this study are as follows: HbcircTAH12-F/R, HbcircARF3-F/R, HbcircTTL1-F/R, HbcircSCSA1-F/R, HbcircARFGAP8-F/R, HbcircFAAH-F/R, HbcircCEMP-F/R, HbcircGMI1-F/R, HbcircSUGP1-F/R, HbcircABP-F/R, HbcircARMSP-F/R, and HbcircSMPD1-F/R, together with the primers for their corresponding linear genes. To explore the effects of HbcircRNAs and their homologous HbLinearRNAs on *H. brasiliensis* resistance to *E. quercicola*, a gene fragment with the same sequence but opposite direction was cloned, and a restriction endonuclease Hindiii site was introduced between the forward and reverse fragments. Finally, this inverted repeat sequence fragment was ligated into the pENTR vector to construct the pENTR-Hb400 vector. Subsequently, the target fragments of HbcircRNAs and HbLinearRNAs were cloned from *H. brasiliensis* cDNA and recombined into the pENTR-Hb400 vector via the restriction endonuclease Hindiii site to generate intermediate vectors. The Gateway™ LR Clonase™ II Enzyme Mix Kit (Thermo Fisher Scientific, Waltham, MA, USA) was then used to clone the target fragments from the pENTR-Hb400-OE vector into the pBA-Flag-Myc4-DC vector for the construction of overexpression vectors. The pHK-35S-amiRNA vector (Zomanbio, Beijing, China) was selected as the silencing vector. A 11 bp sequence on the left and a 10 bp sequence on the right of the back-splice junction (BSJ) of HbcircRNAs were selected, and their reverse complementary sequences were designed and cloned into the pHK-35S-amiRNA vector according to the manufacturer’s instructions (Supplementary Table S1).

### 4.4. CircRNA Validation

Divergent primers and convergent primers were designed separately to verify the authenticity of circRNAs. The extracted total RNA was treated using an RNase R Kit (Beyotime, Shanghai, China). RNase R is a 3′-5′ exoribonuclease that specifically degrades linear RNA molecules but does not digest circular RNAs (circRNAs) due to their lack of free 3′ ends. It is widely used to validate the circular structure of circRNAs and distinguish them from their linear homologous transcripts. PCR validation of circRNAs was performed using RNase R-treated cDNA, RNase R-untreated cDNA, and genomic DNA as templates, respectively. PCR amplification was carried out using Version 2.0 plus dye reagent (Takara, Kusatsu, Shiga, Japan) with the following program: 95 °C for 5 min; 35 cycles of 95 °C for 30 s, 56 °C for 30 s, 68 °C for 60 s/Kb; and a final extension at 68 °C for 10 min. After amplification, the PCR products were purified using the FastPure Gel DNA Extraction Kit (Vazyme, Nanjing, China) prior to sequencing. The authenticity of circRNA back-splicing events was confirmed by verifying the sequence accuracy of the amplified fragments (Supplementary Table S1: Primer sequence).

### 4.5. dsRNA Application

dsRNA fragments were synthesized using the T7 RNAi Transcription Kit (Vazyme). The primer names used in this study are listed below: dsGFP-F, dsGFP-R, RNAi-SCSA1-1, RNAi-SCSA1-2, RNAi-SCSA1-1*, RNAi-SCSA1-2*, RNAi-ARF3-1, RNAi-ARF3-2, RNAi-ARF3-1*, RNAi-ARF3-2*, RNAi-ARFGAP8-1, RNAi-ARFGAP8-2, RNAi-ARFGAP8-1*, RNAi-ARFGAP8-2*, RNAi-FAAH-1, RNAi-FAAH-2, RNAi-FAAH-1*, RNAi-FAAH-2*, RNAi-74-1, RNAi-74-2, RNAi-74-1*, RNAi-74-2*, RNAi-97-1, RNAi-97-2, RNAi-97-1*, RNAi-97-2*, HbLinARF3-RNAi-F, HbLinARF3-RNAi-R, HbLinARFGAP8-RNAi-F, HbLinARFGAP8-RNAi-R, HbLinSCSA1-RNAi-F, HbLinSCSA1-RNAi-R, HbLinFAAH-RNAi-F, HbLinFAAH-RNAi-R, HbLinCEMP-RNAi-F, HbLinCEMP-RNAi-R, HbLinARMSP-RNAi-F, HbLinARMSP-RNAi-R. The specific primer sequences are provided in the RNAi section of Supplementary Table S1. Conidia were collected from 2-week-old *E. quercicola* colonies and suspended in distilled water containing 0.01% Tween-20. The spore concentration was adjusted to 1 × 10^6^ spores/mL using a hemocytometer (Zomanbio, Beijing, China). The 100 ng/μL dsRNA solution was sprayed onto the leaf surfaces of *H. brasiliensis* using a 2 mL spray bottle. After the dsRNA was fully absorbed by the leaves, a 2 μL aliquot of *Erysiphe quercicola* spore suspension was inoculated onto the leaf surfaces. At 48 h post-inoculation, the *H. brasiliensis* seedlings with inoculated leaves were cultured at 23 ± 1 °C with a relative humidity of 70% under a photoperiod of 16 h light/8 h dark. *H. brasiliensis* leaves were collected at 1, 3, 5, and 7 days post-inoculation. Total RNA was extracted, and the transcript levels of target genes were analyzed using qRT-PCR.

### 4.6. qRT-PCR Quantitative Analysis

Bronze-stage leaves inoculated with *E. quercicola* after silencing were collected, and total RNA was extracted and reverse-transcribed into cDNA. qRT-PCR detection was performed using the Taq Pro Universal SYBR qPCR Master Mix Kit (Vazyme, Nanjing, China) with specific primers designed for the relevant genes. The *H. brasiliensis Actin* gene was used as the reference gene to normalize the expression levels. Three biological replicates and three technical replicates were set for the experiment. The relative expression levels of target genes were calculated based on the repeated experimental data, and the qRT-PCR results were quantitatively analyzed using the 2^−ΔΔCt^ method (Supplementary Table S1: Primer sequence).

### 4.7. Agrobacterium-Mediated Transient Gene Expression Assay

The plasmids containing specific expression cassettes were transformed into *Agrobacterium tumefaciens* strain GV3101. *Agrobacterium tumefaciens* cells carrying the target plasmids were cultured, collected, washed, and resuspended in injection buffer containing 1 M MES, 0.1 M AS, and 10 mM MgCl_2_, with the OD_600_ value adjusted to 1.0. The bacterial suspension was injected into the leaves of 4-week-old *Nicotiana benthamiana* using a syringe. Leaf samples were collected at 48 h post-injection for subsequent experiments.

### 4.8. Agrobacterium-Mediated Floral Dip Transformation of Arabidopsis thaliana

The plasmids containing specific expression cassettes were transformed into *Agrobacterium tumefaciens* strain GV3101. *Agrobacterium tumefaciens* cells carrying the target plasmids were cultured, collected, washed, and resuspended in infiltration buffer containing 5% sucrose and 0.02% Silwet-L77, with the bacterial concentration adjusted to an OD_600_ value of 1.5–2.0 [57]. *Arabidopsis* plants grown at 22 ± 1 °C, under a 16 h light/8 h dark photoperiod and 70% relative humidity, had their inflorescences fully immersed in the bacterial suspension for 30 s, followed by incubation under humid and dark conditions for 24 h. The inflorescences of *Arabidopsis thaliana* were completely immersed in the bacterial suspension for 30 s, followed by cultivation under moist and dark conditions for 24 h. The harvested *Arabidopsis thaliana* seeds were subjected to vernalization at 4 °C. For surface sterilization, the *Arabidopsis thaliana* seeds were soaked in 70% ethanol for 1 min and then in sodium hypochlorite solution (water: sodium hypochlorite = 4:1) for 15 min with continuous shaking, followed by washing three times with ddH_2_O. MS medium containing the corresponding antibiotics was prepared, and the seeds were sown evenly on the medium surface. The plates were placed in a light incubator and cultured at 22 ± 1 °C under a photoperiod of 16 h light/8 h dark. The normally germinated *Arabidopsis thaliana* seedlings were transplanted for further cultivation.

### 4.9. Lesion Area Detection

Conidia were collected from 2-week-old *E. quercicola* colonies and suspended in sterile distilled water containing 0.01% Tween-20. The spore concentration was adjusted to 1 × 10^6^ spores/mL using a hemocytometer. A 2 μL aliquot of *E. quercicola* spore suspension, a mixture of GFP-dsRNA and *E. quercicola* spore suspension, a mixture of 100 ng/μL dsHbcircRNAs and *E. quercicola* spore suspension, or a mixture of 100 ng/μL dsHbLinearRNAs and *E. quercicola* spore suspension was inoculated onto the leaf surfaces of *H. brasiliensis*, respectively. At 48 h post-inoculation, the *H. brasiliensis* seedlings with inoculated leaves were cultured in an experimental greenhouse to promote the colonization and growth of *E. quercicola*. The greenhouse conditions were set as 23 ± 1 °C with a relative humidity of 70% under a photoperiod of 16 h light/8 h dark. At 7 days post-inoculation, the infection phenotypes of leaves were photographed using a digital camera under uniform light conditions, and the lesion areas were measured and calculated using ImageJ software (version 1.54r).

### 4.10. Determination of Reactive Oxygen Species (ROS) and Callose

A 0.1% DAB staining solution was prepared by adding 0.05 g of DAB to 50 mL of ddH_2_O under light-proof conditions, and the mixture was vortexed until DAB was completely dissolved. *Hevea brasiliensis* leaves or *Arabidopsis thaliana* leaf tissues were immersed in 20 mL of 0.1% DAB staining solution and stained overnight on a shaker, with the entire process performed in the dark. The staining solution was discarded, and 40 mL of decolorizing solution (ethanol:acetic acid = 3:1) was added. The samples were incubated in a boiling water bath for 20 min to rinse off chlorophyll. The decolorized leaf tissues were kept moist in ddH_2_O, and the accumulation of ROS in *H. brasiliensis* leaves or *Arabidopsis thaliana* leaves was observed under a bright-field microscope(Nikon Corporation, Tokyo, Japan). *Hevea brasiliensis* leaves or *Arabidopsis thaliana* leaf tissues inoculated with *E. quercicola* were decolorized overnight at room temperature in 40 mL of decolorizing solution (ethanol:acetic acid = 3:1) until chlorophyll was completely removed. The decolorizing solution was discarded, and 30 mL of ddH_2_O was added to wash the samples at room temperature for 30 min. Subsequently, the leaf samples were immersed in aniline blue solution (0.1% aniline blue, 67 mM Na_2_HPO_4_, pH = 12.0) for 2 h. The leaves treated with aniline blue solution were observed using a fluorescence microscope (Nikon Corporation, Tokyo, Japan).

### 4.11. Protein Extraction, Western Blot Analysis and Coomassie Brilliant Blue Staining

Western blot analysis was performed on the obtained HbLinearRNA-OE transgenic *Arabidopsis* lines. Frozen plant samples were fully ground into powder with liquid nitrogen, and 200 μL of protein extraction buffer (CWBIO, Taizhou, China) was added rapidly. The mixture was vortexed vigorously to mix well and placed on ice for 30 min, with vortexing every 5 min to ensure sufficient protein lysis. The samples were centrifuged at 12,000 rpm for 15 min at 4 °C, and the supernatant was carefully aspirated and centrifuged again at 12,000 rpm for 15 min at 4 °C. The resulting supernatant was collected as the total protein extract. A 100 μL aliquot of the supernatant was mixed with 25 μL of 5× SDS-PAGE protein loading buffer (Sevier, Wuhan, China) and incubated in a boiling water bath for 10 min. A 1.5% SDS-PAGE gel was prepared, and after electrophoresis, the proteins were transferred onto a membrane. The membrane was placed in 5% skim milk blocking solution and blocked at 4 °C for 5 h. The membrane was washed three times with 1× PBST buffer (containing 100 mL of 1× PBS and 0.05% Tween-20) for 10 min each time. Then, the membrane was incubated overnight at 4 °C with Flag primary antibody incubation solution (containing 2 μL of Flag primary antibody and 1 g of skim milk dissolved in 20 mL of 1× PBST buffer). After washing three times with 1× PBST buffer, the membrane was incubated with HRP-conjugated mouse secondary antibody incubation solution (containing 2 μL of HRP-conjugated mouse secondary antibody and 1 g of skim milk dissolved in 20 mL of 1× PBST buffer) at 4 °C for 45 min. After incubation, the membrane was washed three times with 1× PBST buffer again. The target protein bands were detected, observed, and recorded using a chemiluminescence imaging system.

After SDS-PAGE electrophoresis, the gel was removed and placed in an appropriate amount of Coomassie Brilliant Blue staining solution (BL605A, White Shark Biotech, Hefei, China), ensuring that the staining solution completely covered the gel. The gel was stained with gentle shaking at room temperature for 1 h. After staining, the staining solution was discarded, and an appropriate amount of Coomassie Brilliant Blue decolorizing solution (BL606A, White Shark Biotech, Hefei, China) was added. The gel was decolorized with shaking for 12 h until the blue background of the gel was basically removed and the protein bands were clearly visible.

### 4.12. RNA-Seq Library Construction and Sequencing Analysis

RNA-Seq analysis was performed for each experimental group. Total RNA was extracted from exosomes using the RNAiso Plus method [58], and the extracted RNA was sampled for quality inspection. The RNA concentration, purity, and integrity were detected using an Agilent 2100 Bioanalyzer(Agilent Technologies, Santa Clara, CA, USA). RNA samples with a concentration of not less than 200 ng/μL, an OD_260_/OD_280_ ratio greater than 1.80, and an RIN value > 7 were used for subsequent experiments. After passing quality assessment, sequencing libraries were constructed using the GENESEED^®^ RNA-seq Library Prep Kit for Illumina (Guangzhou Geneseed Biotech Co., Ltd., Guangzhou, China), with the target insert size set to 250–450 bp as recommended in the kit protocol. After library construction, preliminary quantification was performed using a Qubit 3.0 Fluorometer (Thermo Fisher Scientific, Waltham, MA, USA), and then the size range of the libraries was detected using an Agilent 2100 Bioanalyzer. After confirming that the size of the inserted target fragments met the expectations, the effective concentration of the libraries was accurately quantified using the Q-PCR method (effective library concentration > 3 nM) to ensure library quality. After passing the library inspection, different libraries were pooled according to the effective concentration and the required amount of target off-machine data, and sequenced on the NovaSeq X Plus platform (Illumina Inc., San Diego, CA, USA) in the PE150 mode, with a sequencing data volume of 10 G. FastQC software (version 0.11.9) was used for quality control of the sequencing data. Raw reads were filtered to remove adapter sequences, sequences with a high content of N, and low-quality reads to obtain high-quality data (Clean reads). The Clean reads were then mapped to the reference genome, and the mapping results were used for subsequent circRNA identification. According to the results provided by Guangzhou Giesee Bio-Tech Co., Ltd., the total output of RNA-seq data was 10 Gb, which is sufficient for the subsequent bioinformatic analysis. A total of 7010 transcripts were detected before differential expression analysis. For the inter-group comparative analysis, lowly expressed circRNAs were filtered by the company based on TMM normalization: only circRNAs with CPM > 0.1 in at least half of the samples were retained. Differential expression analysis of circRNAs was then performed using the edgeR package(version 3.44.0), with thresholds: fold-change ≥ 1.5, *p* < 0.05, FDR < 1. Under these criteria: 975 transcripts were identified in the 1 vs. 0 comparison; 876 transcripts were identified in the 3 vs. 0 comparison.

### 4.13. Analysis of CircRNA Expression Levels

The reads were first mapped to the latest UCSC transcript set using Bowtie2 version 2.1.0 [59], and the gene expression levels were estimated using RSEM v1.2.15 [60]. For lncRNA expression analysis, the transcript set from Lncipedia (http://www.lncipedia.org, accessed on 15 June 2024) (https://www.omicshare.com/tools/ accessed on 15 December 2025) was used. The gene expression levels were normalized using the trimmed mean of M-values (TMM) method. Differentially expressed genes were identified using the edgeR program [61]. Genes with altered expression showing a *p*-value < 0.05 and a fold change > 1.5 were considered differentially expressed. Fischer’s exact test with the false discovery rate (FDR) option was used to calculate the significance of the canonical pathways.

For circRNA expression analysis, the reads were mapped to the genome using STAR [62], and DCC [63] was used to identify circRNAs and estimate their expression levels. The gene expression levels were normalized using the TMM method. Differentially expressed genes were identified using the edgeR program. miRanda [64] was used to predict the miRNA targets of circRNAs. R software (version 4.3.1) was used to generate the figures. The detailed data of circRNAs in this study are presented in Supplementary Table S2.

### 4.14. Functional Correlation Analysis of CircRNAs and LinearRNAs

The Sankey diagram for the functional correlation analysis of circRNAs and linearRNAs was generated using the OmicShare Cloud Tools platform (https://www.omicshare.com/tools/ (accessed on 9 February 2026)).

## Data Availability

The authors confirm that the data supporting the findings of this study are available. The raw sequencing data have been deposited to NCBI under the following accession numbers. HbcircTAF12: LG02_92295427_92298349_+. LOC110646615. HbcircARF3: LG04_9262444_9263092_-. LOC110650964. HbcircTTL1: LG09_58585131_58586854_-. LOC110670358. HbcricSCSA1: LG03_62633794_62635128_-. LOC110657393. HbcircARFGAP8: LG09_17800838_17801270_-. LOC110656620. HbcircFAAH: LG16_40914365_40914884_-, LOC110644151. HbcircSMPD1: LG18_6118275_6120184_-. LOC110646088. HbcircCEMP: LG05_78561612_78562233_-. LOC110663331. HbcircABP: LG01_43254529_43256334_-. LOC110639187 HbcircARMSP: LG02_87009198_87014455_-. LOC110663402. HbcircGMI1: LG10_66804109_66807728_-. LOC110633695. HbcircSUGP1: LG15_1422229_1423458_-, LOC110643685.

## Supplementary Materials

The following supporting information can be downloaded at: https://www.mdpi.com/article/10.3390/plants15071068/s1. Figure S1: Phenotypic and molecular validation of *E. quercicola* colonization on *H. brasiliensis* bronze-stage leaves; Figure S2: Expression dynamics of HbcircRNAs and their homologous HbLinearRNAs under different silencing treatments; Figure S3 RNA Silencing targeting HbcircRNAs and their homologous HbLinearRNAs participates in ROS accumula-tion and callose deposition during powdery mildew infection in *H. brasiliensis* leaves; Figure S4: Identification of trans-genic *Arabidopsis* lines expressing HbcircRNAs and their homologous HbLinearRNAs; Table S1: Primer sequence; Table S2: *Hevea brasiliensis* circRNA data.

## Abbreviations

The following abbreviations are used in this manuscript:

SIGS	Spray-induced gene silencing
RNase R	Ribonuclease R
dpi	Days post-inoculation
DCC	Discovery of Circular RNAs from High-throughput Sequencing Data
