# Supplementary material for: Differential Roles of Circular RNAs and Their Homologous Linear RNAs in Hevea brasiliensis Immunity Against Erysiphe quercicola
Source: Plants (Basel). 2026 Mar 31;15(7):1068. doi: 10.3390/plants15071068 (PMC13074488; doi:10.3390/plants15071068)
Supplement: Supplementary file 1 [file plants-15-01068-s001.zip › Supplementary figure.pdf]

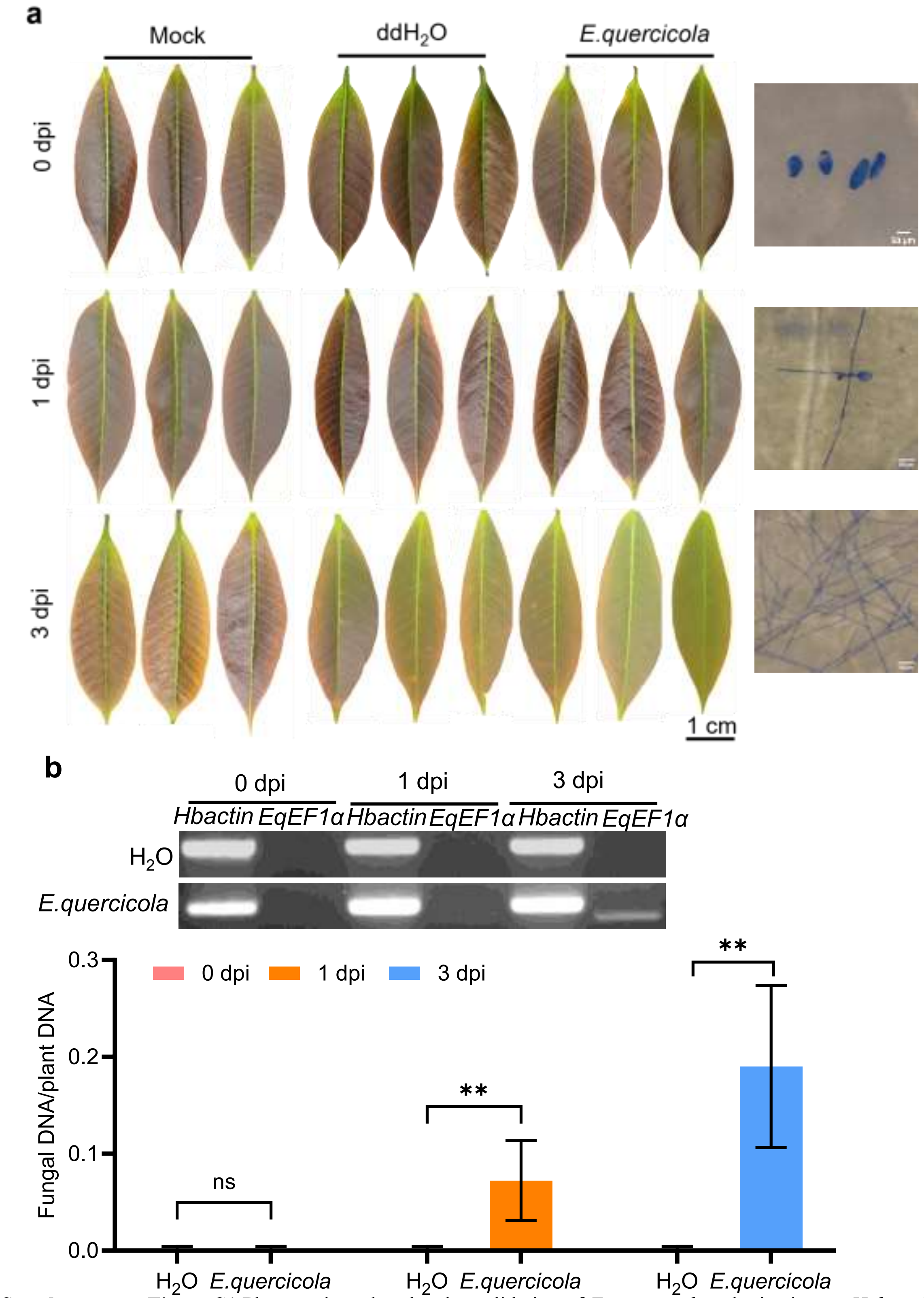


**Supplementary Figure S1** Phenotypic and molecular validation of *E. quercicola* colonization on *H. brasiliensis* bronze-stage leaves (a) Phenotypic observation of rubber tree bronze-stage leaves under different treatments at 0, 1, and 3 days post inoculation (dpi). Treatments included blank control (Mock), water control ($ddH_2O$), and *E. quercicola* inoculation. Scale bar = 1 cm. Visible phenotypic alterations were observed in *E. quercicola*-inoculated leaves at 3 dpi, whereas no obvious changes occurred in Mock or $ddH_2O$ groups. Microscopic observation of different treatments on rubber tree leaves at the bronze stage at 0, 1, and 3 days post-inoculation (dpi).Scale bar = 50 µm. (b) Molecular validation of *E. quercicola* colonization. PCR amplification results of host reference gene *Hbactin* (internal control for plant tissue) and pathogen reference gene *EqEF1α* (internal control for *E. quercicola*) in $ddH_2O$-treated and *E. quercicola*-inoculated samples at 0, 1, and 3 dpi. Quantitative analysis of the fungal DNA/plant DNA ratio (normalized to *EqEF1α*/*Hbactin*). Statistical significance was determined using Student's t-test: "ns" indicates no significant difference ($P > 0.05$), and "**" indicates extremely significant difference ($P < 0.01$). The results showed that *E. quercicola*-specific DNA was undetectable in $ddH_2O$-treated samples at all time points, while it was significantly accumulated in inoculated samples at 1 dpi and 3 dpi, confirming successful colonization of *E. quercicola* on rubber tree leaves. Each bar represents the mean ± SEM from three independent experiments. These experiments wereperformed with three independent biological replicates (n = 9) with similar results.

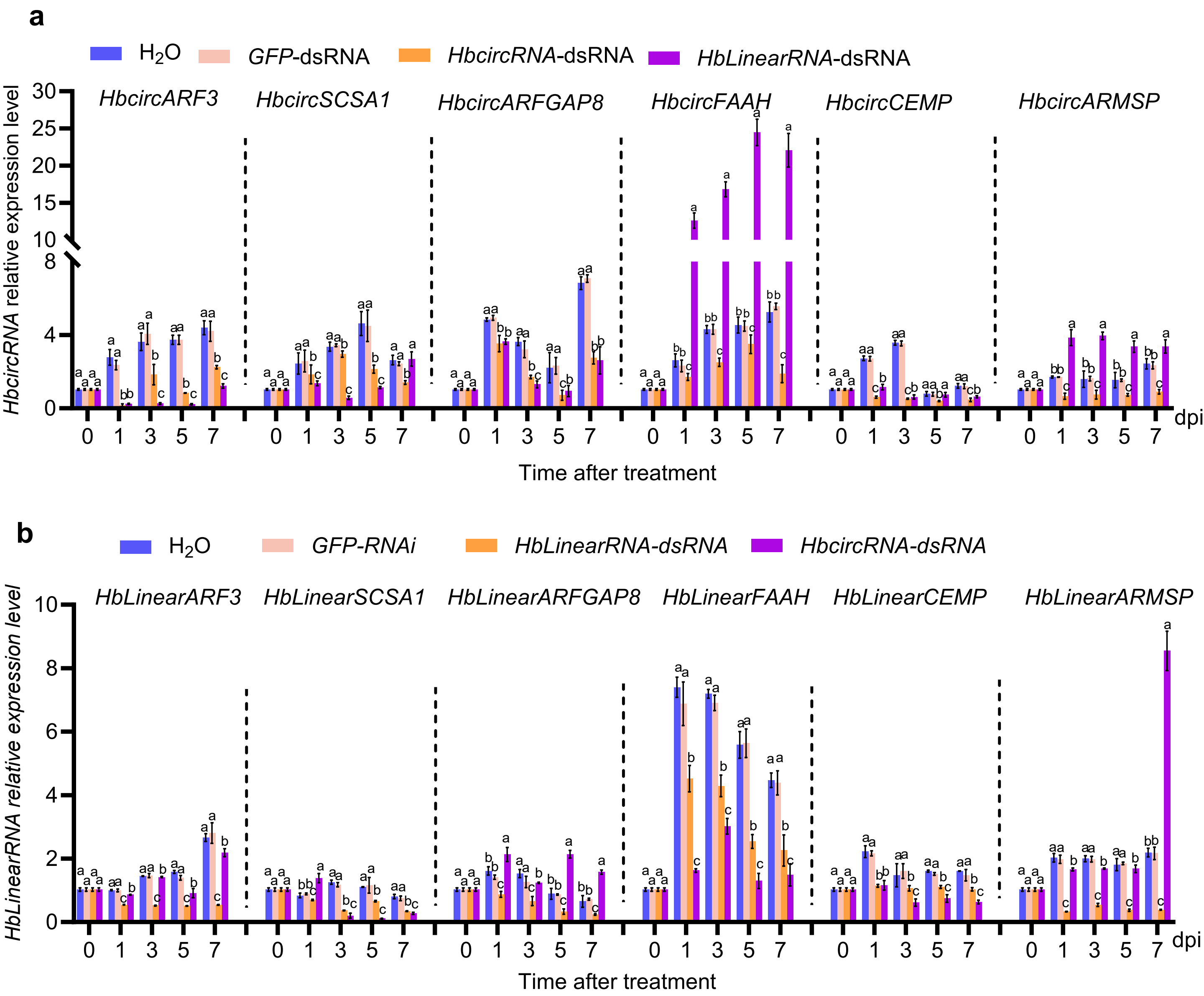


**Supplementary Figure S2** Expression dynamics of HbcircRNAs and their homologous HbLinearRNAs under different silencing treatments

(A) After inoculation with powdery mildew, total RNA was collected from rubber tree leaves at 0, 1, 3, 5, and 7 days post inoculation (dpi) following *HbcircRNAs* silencing, and the transcript levels of *HbcircRNAs* and their homologous *HbLinearRNAs* were detected by qRT-PCR. The rubber tree *actin* gene was used as an internal control. Bar charts show the mean values and standard deviations with three biological replicates (n=3 per replicate). One-way ANOVA followed by Tukey's test was used for statistical analysis, and different letters indicate significant differences (P < 0.01).

(B) After inoculation with powdery mildew, total RNA was collected from rubber tree leaves at 0, 1, 3, 5, and 7 days post inoculation (dpi) following *HbLinearRNAs* silencing, and the transcript levels of *HbcircRNAs* and their homologous *HbLinearRNAs* were detected by qRT-PCR. The rubber tree *actin* gene was used as an internal control. Bar charts show the mean values and standard deviations with three biological replicates (n=3 per replicate). One-way ANOVA followed by Tukey's test was used for statistical analysis, and different letters indicate significant differences (P < 0.01).

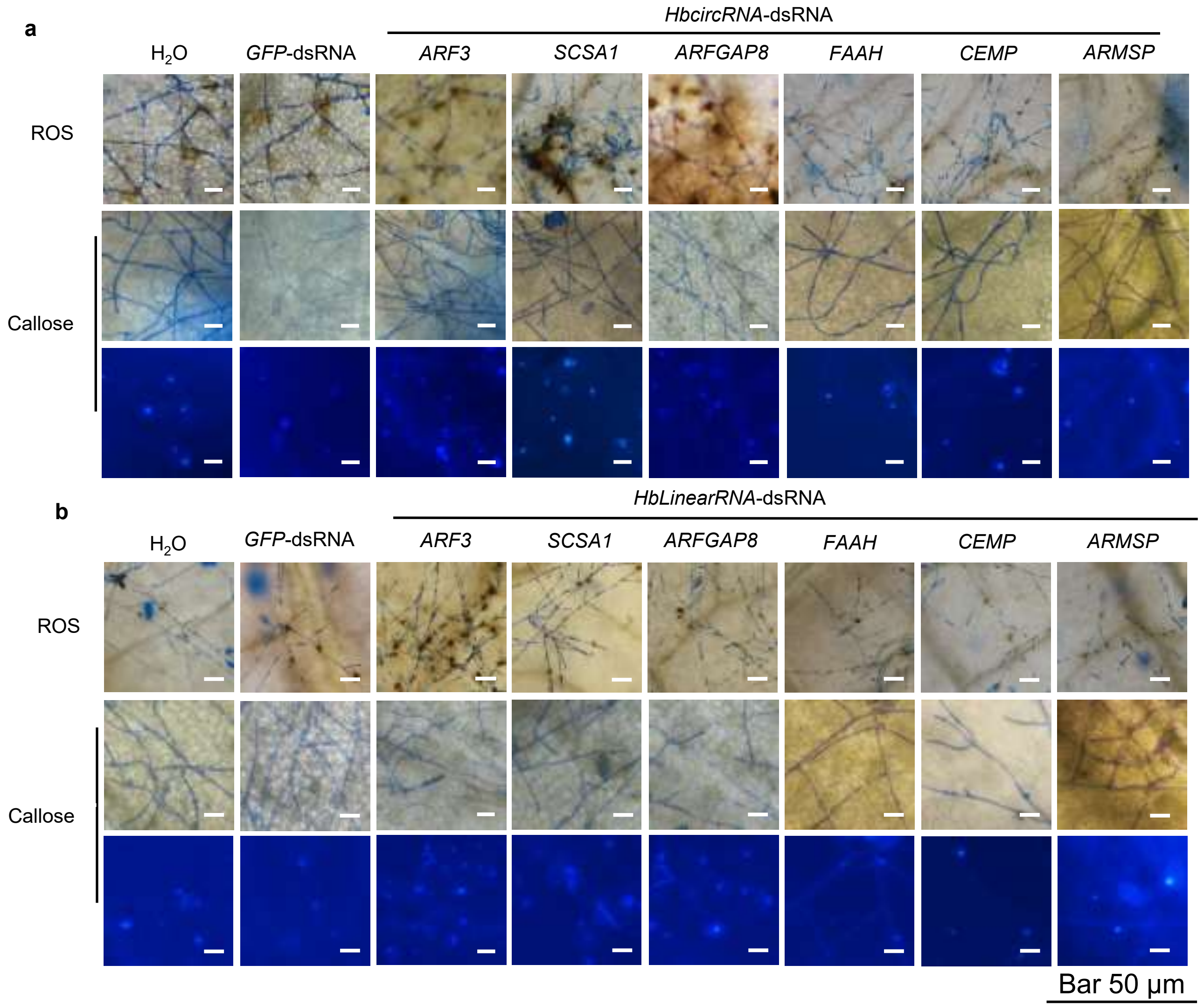


**Supplementary Figure S3** RNA Silencing Targeting HbcircRNAs and Their Homologous HbLinearRNAs Participates in ROS Accumulation and Callose Deposition During Powdery Mildew Infection in *H. brasiliensis* Leaves. (a) DAB and aniline blue staining show reactive oxygen species (ROS) accumulation and callose deposition, respectively, at the infection sites of H. brasiliensis leaves after HbcircRNAs silencing. Scale bar, 50 μm. (b) DAB and aniline blue staining show ROS accumulation and callose deposition, respectively, at the infection sites of H. brasiliensis leaves after HbLinearRNAs silencing. Scale bar, 50 μm.

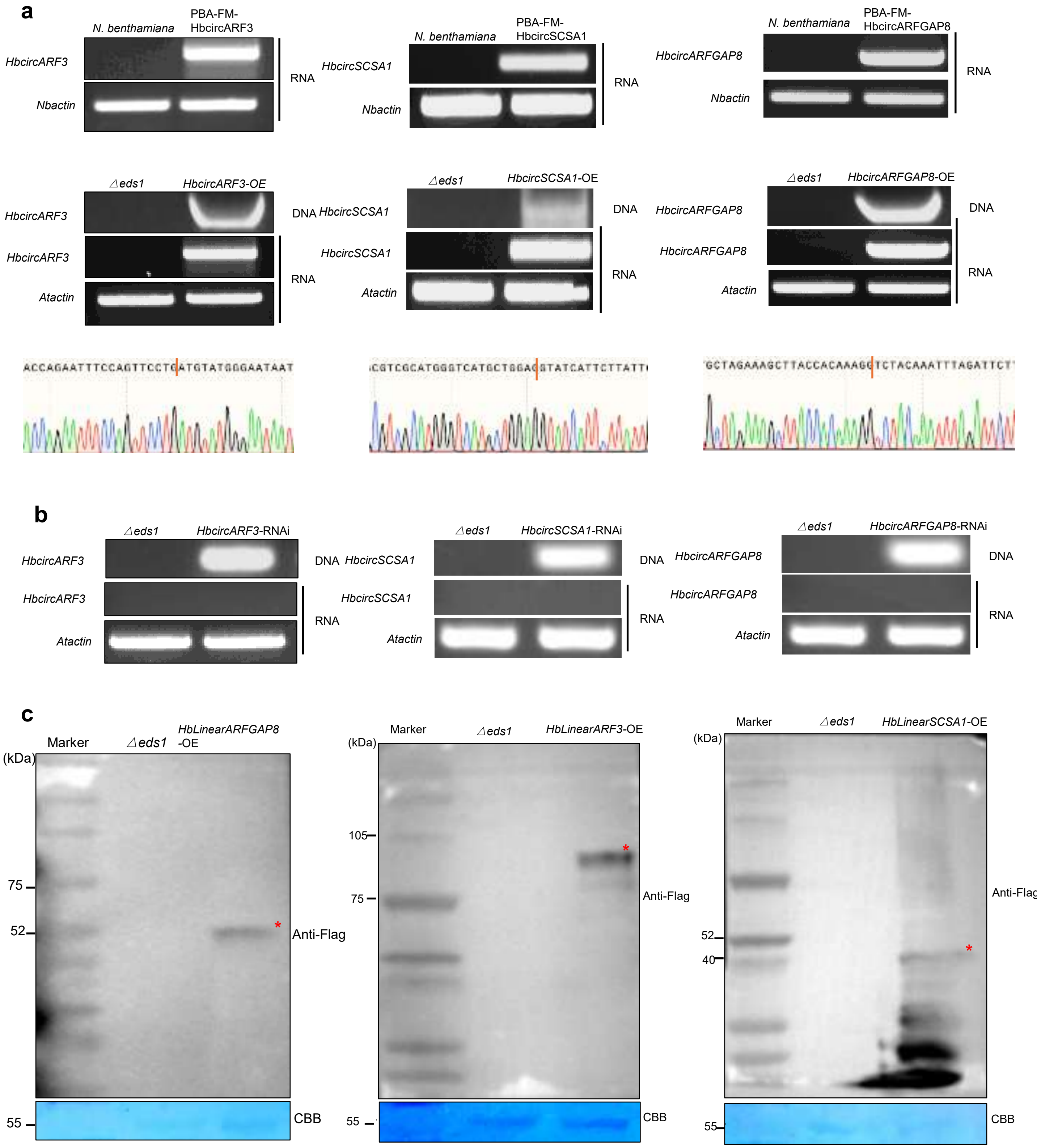


**Supplementary Figure S4** Identification of transgenic *Arabidopsis* lines expressing *HbcircRNAs* and their homologous *HbLinearRNAs*

(a) Validation of *HbcircRNAs* overexpression lines in tobacco and *Δeds1*, respectively. RNA was extracted from tobacco to verify the overexpression constructs. Identification was performed at both DNA and RNA levels in *Δeds1*. Sanger sequencing validation of the circRNA back-splicing site. The orange vertical bar denotes the back-splicing site.

(b) Validation of *HbcircRNAs* silencing lines in *Δeds1*. Identification was performed at both DNA and RNA levels in *Δeds1*.

(c) Western blot analysis of total proteins extracted from *Arabidopsis* transgenic lines expressing *HbLinearRNAs*. The protein bands of interest, indicated by red asterisks, were detected using anti-Flag antibody. Coomassie brilliant blue staining of the PVDF membrane was used as a loading control.
