# Supplementary material for: Differential Roles of Circular RNAs and Their Homologous Linear RNAs in Hevea brasiliensis Immunity Against Erysiphe quercicola
Source: Plants (Basel). 2026 Mar 31;15(7):1068. doi: 10.3390/plants15071068 (PMC13074488; doi:10.3390/plants15071068)
Supplement: Supplementary file 1 [file plants-15-01068-s001.zip › Supplementary tab.pdf]

Supplementary table S1

Primer	Primer sequence (5’-3’)	Work
HbcircTAH12-F	CAAAAGACATACTTCTACATCTTG	Validation of HbcircRNAs
HbcircTAF12-R	CTTCCAATGTATCAGATTTTCGATG	
HbcircARF3-R	GAAACCCTGCTGTGCCTG	
HbcircARF3-F	TCCTTGGGAAATTGAGCCATC	
HbcircTTL1-R	GGCATGCTTCAGCATCTTCAAG	
HbcircTTL1-F	GCAAATGCTGAAAATGTGAGGATC	
HbcircSCSA1-R	GAGTTCCACTTTCCTTAATCAGAGC	
HbcircSCSA1-F	AAAGCCTATTGTTGCATTTATAGCTG	
HbcircARFGAP8-R	CTGACTGAGAAGCAACAGGCAA	
HbCircARFGAP8-F	CCCAAGTGTCCAATGGACTTC	
HbcircFAAH-R	CTAGAATAATCCATTATTGTCCAACGACG	
HbcircFAAH-F	AGCCTATAATTCTGGACAAATAACTCCC	
HbcircCEMP-F	TGGCAGACCTTGTTGGAAGTA	
HbcircCEMP-R	ACCAGATTCAGAATGATACCCT	
HbcircGMI1-F	TCATGTCATACTACAATCACCGTTAC	
HbcircGMI1-R	TCTAAATGTCCAGGAGTGACTTTAC	
HbcircSUGP1-F	AACCCACGAATTACTGGTCTAG	
HbcircSUGP1-R	TTTAATTGTCGATCGCAAGATTATTACTC	
HbcircABP-F	AATATACTGGGAGGATTCACCTG	
HbcircABP-R	TACTTACCTCAAAGCGCAGAG	
HbcircARMSP-F	GGGAGAGTAAATCCTGCACG	
HbcircARMSP-R	ATCTGGACAATAAGCCCTTGC	
HbcircSMPD1-F	TCTTGCATGAGAATTGGAACAAAG	
HbcircSMPD1-R	AAGATCCATCAGCATCACTCTC	
HblineTAF12-F	ATCGACCCTTATGAGAAATTGGAT	
HblineTAF12-R	TGCTGCGAGACGTTCCTT	
HblineARF3-F	ATGTATGGGAATAATAACTGGAATTAGTG	
HblineARF3-R	CAGGAACTGGAAATTCTGGTTTTGA	
HblineTTL1-F	GTTAGGACAGGTTGAGAATGCC	
HblineTTL1-R	CAGTGTATCTCTCAGATTTGAAGAG	
HblineSCSA1-F	GTATCATTCTTATTGGTGAAATAGGAGG	
HblineSCSA1-R	CTCCAGCATGACCCATGC	
HblineARFGAP8-F	GTCTACAAATTTAGATTCTTGGAGTCATG	
HblineARFGAP8-R	CTTTGTGGTAAGCTTTCTAGCAC	
HblineFAAH-F	CTTGTTTCGAATGCAGAGCT	
HblineFAAH-R	CTGTTTGATACCTTTGAGTTGATTCA	
HblineCEMP-F	GTAGCTTTGCTGAAATTTACTGGATA	
HblineCEMP-R	ACGCATGCGTCAATAACAAC	
HblineABP-F	GTAGAAAAGGCTAGTGGTGTACTTG	

HblineABP-R	CTCAGTACTGTGGTTAATATTT	
HblineARMSP-F	AACTGGAACTCCTTGATTGG	
HblineARMSP-R	CATGAGAAATGCCTTGGATGG	
dsGFP-F	TAATACGACTCACTATAGGGATGGTGAGCAAGGGCGAGGAGC	RNAi
dsGFP-R	TAATACGACTCACTATAGGGGGTGCGCTCCTGGACGTAG	
RNAi-SCSA1-1	GATCACTAATACGACTCACTATAGGGctccagcatgaa	
RNAi-SCSA1-2	GATCACTAATACGACTCACTATAGGGaagaatgatacaa	
RNAi-SCSA1-1*	ttcatgctggagCCCTATAGTGAGTCGTATTAGTGATC	
RNAi-SCSA1-2*	ttgtatcattcttCCCTATAGTGAGTCGTATTAGTGATC	
RNAi-ARF3-1	GATCACTAATACGACTCACTATAGGGcaggaactggaa	
RNAi-ARF3-2	GATCACTAATACGACTCACTATAGGGttcccatacataa	
RNAi-ARF3-1*	ttccagttcctgCCCTATAGTGAGTCGTATTAGTGATC	
RNAi-ARF3-2*	ttatgtatgggaaCCCTATAGTGAGTCGTATTAGTGATC	
RNAi-ARFGAP8-1	GATCACTAATACGACTCACTATAGGGctttgtggtaaa	
RNAi-ARFGAP8-2	GATCACTAATACGACTCACTATAGGGaatttgtagacaa	
RNAi-ARFGAP8-1*	tttaccacaaagCCCTATAGTGAGTCGTATTAGTGATC	
RNAi-ARFGAP8-2*	ttgtctacaaattCCCTATAGTGAGTCGTATTAGTGATC	
RNAi-FAAH-1	GATCACTAATACGACTCACTATAGGG tgtttgatacaa	
RNAi-FAAH-2	GATCACTAATACGACTCACTATAGGG tcgaaacaagcaa	
RNAi-FAAH-1*	ttgtatcaaacaCCCTATAGTGAGTCGTATTAGTGATC	
RNAi-FAAH-2*	ttgcttgtttcgaCCCTATAGTGAGTCGTATTAGTGATC	
RNAi-74-1	GATCACTAATACGACTCACTATAGGGAAAGCTTTGGTT	
RNAi-74-2	GATCACTAATACGACTCACTATAGGGGTAGCTTTGCTTT	
RNAi-74-1*	AACCAAAGCTTTCCCTATAGTGAGTCGTATTAGTGATC	
RNAi-74-2*	AAAGCAAAGCTACCCCTATAGTGAGTCGTATTAGTGATC	
RNAi-97-1	GATCACTAATACGACTCACTATAGGGATTTCTCATGTT	
RNAi-97-2	GATCACTAATACGACTCACTATAGGGGTCCAAAACTGTT	
RNAi-97-1*	AACATGAGAAATCCCTATAGTGAGTCGTATTAGTGATC	
RNAi-97-2*	AACAGTTTTGGACCCCTATAGTGAGTCGTATTAGTGATC	
HbLinARF3-RNAi-F	TAATACGACTCACTATAGGGTCTTGTGGGCCCATTCAACA	
HbLinARF3-RNAi-R	TAATACGACTCACTATAGGGTCCTTTGGTACAGTTGCTCCC	
HbLinARFGAP8-RNAi-F	TAATACGACTCACTATAGGGGGCTGGAGGTCCGCAAGTGATT	
HbLinARFGAP8-RNAi-R	TAATACGACTCACTATAGGGGGCAACATCGGTGGCTTTGCTC	
HbLinSCSA1-RNAi-F	TAATACGACTCACTATAGGGGGCATTTGCCAAGGCATCACCG	
HbLinSCSA1-RNAi-R	TAATACGACTCACTATAGGGGGGTGGGACAGAACACTTGGGA	
HbLinFAAH-RNAi-F	TAATACGACTCACTATAGGGAAGCTCCACATTTGACCGGA	
HbLinFAAH-RNAi-R	TAATACGACTCACTATAGGGCTGAGTCCCCATTCCAGCAG	
HbLinCEMP-RNAi-F	TAATACGACTCACTATAGGGGCGTCACCCAATTGTTTGCT	
HbLinCEMP-RNAi-R	TAATACGACTCACTATAGGGTCCCTCAACTCCACAATGGC	
HbLinARMSP-RNAi-F	TAATACGACTCACTATAGGGACCGTCTCGCCTTTCAATGT	
HbLinARMSP-RNAi-R	TAATACGACTCACTATAGGGAAGGCCGACTCTTTCTGCTC	
RT-circARFGAP8-F	AGCTTACCACAAAGGTCTACAA	qRT-PCR

RT-circARFGAP8-R	CAACAGGCGATGATGGCAAG	
RT-circFAAH-F	ATGCGGTGGCTGAACGATTA	
RT-circFAAH-R	TCGAAACAAGCTGTTTGATACCT	
RT-circSCSA1-F	GCATTTATAGCTGGACTAACTGCTC	
RT-circSCSA1-R	ACCAATAAGAATGATACCTCCAGCA	
RT-circARF3-F	TCCAGTTCCTGATGTATGGGA	
RT-circARF3-R	CCCACCTTACCACAAGGCAT	
RT-circCEMP-F	TGGCAGACCTTGTTGGAAGTA	
RT-circCEMP-R	ACCAGATTCAGAATGATACCCT	
RT-circARMSP-F	GGGAGAGTAAATCCTGCACG	
RT-circARMSP-R	ATCTGGACAATAAGCCCTTGC	
RT-LinARFGAP8-F	GTTGCTTCTCAGTCAGCCCA	
RT-LinARFGAP8-R	GCTTTTGGAGTGGTGGTGTT	
RT-LinFAAH-F	AAGCTCCACATTTGACCGGA	
RT-LinFAAH-R	CTGAGTCCCCATTCCAGCAG	
RT-LinSCSA1-F	GGCAAGAACGGCACTTTTCA	
RT-LinSCSA1-R	CAGTTGTTTGAAAGACCATGTCA	
RT-LinARF3-F	TCTTGTGGGCCCATTCAACA	
RT-LinARF3-R	TCCTTTGGTACAGTTGCTCCC	
RT-LinCEMP-F	GCGTCACCCAATTGTTTGCT	
RT-LinCEMP-R	TCCCTCAACTCCACAATGGC	
RT-LinARMSP-F	ACCGTCTCGCCTTTCAATGT	
RT-LinARMSP-R	AAGGCCGACTCTTTCTGCTC	
RT-SNAP33-F	ATGGCTGCCTGAAGATTGCT	
RT-SNAP33-R	AGGGCCTGTAATTGGACGAT	
RT-SEC22-F	GGATGATGGCCGTGACTTGA	
RT-SEC22-R	ATCTGTGCCCCATTGACACG	
RT-SAUR72-F	CGACTCTTCCCAGTACAGCC	
RT-SAUR72-R	GAAGATCGGGTGGTTCAGCA	
RT-PIN1-F	CGATCAGGTCAGGGCATCTC	
RT-PIN1-R	CTTCTGCATCTGGCCTGGAC	
RT-MYC2-F	GAAGAACCCAACTGAACGGC	
RT-MYC2-R	GATTGTCGGTGACGCTTGTA	
RT-EXO70B1-F	GGATGTCGCCGAGAAAGTCA	
RT-EXO70B1-R	TCCGTACAACATGTCCGCAT	
RT-COI1-F	GGGTGCAAGGTTACAGAGCA	
RT-COI1-R	CCACCAAATCCAAGGGGACA	
RT-AOS-F	CAACATGCCTCCTGGTCCTT	
RT-AOS-R	AGTTGGGTATGCTTCGGCTC	
RT-atp9-F	GCTCATGTTAGCATCGCCAC	
RT-atp9-R	ATGCTAAGTCAGGGGGAGGT	
RT-nad1-F	GTTCAGATCCTCTACCGCCG	
RT-nad1-R	ACCTGATAATGGCAAGGGGC	

RT-NADH-F	CTCGGCCAATTACTCGGGAT	
RT-NADH-R	GCGATACCCAAAGCTCCCAT	
RT-PIN3-F	CAAGGTCCGAGGATGAGCAG	
RT-PIN3-R	TTCAAATTTTGGGCCGCCTG	
PENTR-F	TCGGTGAACGCTCTCCTGAGTAGGACAAAT	Plasmid construction
PENTR-R	CCTCGACGTTTCCCGTTGAATATGGCTCAT	
PBA-FM-DC-F	TACAGTCTCAGAAGACCAAAGG	
Hb400-F	CGACTGGATCCCCAATACTGTATAGAAGAGAATGACCAAAAT GAACAT	
Hb400-R	GGCCGCGAATTCGGTACCGGATCCCCAATACGTATAGAAGAG AATGACCAAAATGAACAT	
Hb400-Hindiii-R	CCTCCTGTATTGCGTTAAGCTTAACGCAATACAGGAGGAAA	
HbcARF3-F1	TCCTGTATTGCGTTAAGCTTGTTTGACATACTCCACTTGCA	
HbcARF3-F2	TGAAATATTGACTAAATTTCTTTGGCAGATGTATGGGAATAATA ACTGGAATTAGTG	
HbcARF3-F3	ACCAGAATTTCCAGTTCCTGGTATGTTCTGCATTTTCAAGAAA C	
HbcARF3-R1	CACTAATTCCAGTTATTATTCCCATACATCTGCCAAAGAAATTT AGTCAATATTTCA	
HbcARF3-R2	GTTTCTTGAAAATGCAGAACATACCAGGAACTGGAAATTCTG GT	
HbcARF3-R3	TCCTGTATTGCGTTAAGCTTAACAAATAAAGACAAGTCAACA CA	
HbcARFGAP8-F1	TCCTGTATTGCGTTAAGCTTCGGCTTAGTACTCAAAACTTT	
HbcARFGAP8-F2	TCTGTTCATAAAAATTATTTATTTTATTCATGTAGGTCTACAAAT TTAGATTCTTGGAGT	
HbcARFGAP8-F3	GCTAGAAAGCTTACCACAAAGGTAGGATTTTACTTACATTTAT GAATGAATT	
HbcARFGAP8-R1	ACTCCAAGAATCTAAATTTGTAGACCTACATGAATAAAATAAA TAATTTTTATGAACAGA	
HbcARFGAP8-R2	AATTCATTCATAAATGTAAGTAAAATCCTACCTTTGTGGTAAGC TTTCTAGC	
HbcARFGAP8-R3	TCCTGTATTGCGTTAAGCTTAAAATTGAACAGAAAATCACTTT AAGG	
HbcSCSA1-F1	TCCTGTATTGCGTTAAGCTTTTGGGTGCTGCTCAAC	
HbcSCSA1-F2	GATAATGTGAATTGCATTGCAGGTATCATTCTTATTGGTGAAAT AGGAG	
HbcSCSA1-F3	ATGGGTCATGCTGGAGGTATTGTATTATCATCTAATATTATCATC TAATTTT	
HbcSCSA1-R1	CTCCTATTTCACCAATAAGAATGATACCTGCAATGCAATTCAC ATTATC	
HbcSCSA1-R2	AAAATTAGATGATAATATTAGATGATAATACAATACCTCCAGCA TGACCCAT	

HbcSCSA1-R3	TCCTGTATTGCGTTAAGCTTGACTAGCAGAGAAGTGCAG	
OELinearARF3-F	ATGTTGGGTATCATAGATCTTAATACCA	
OELinearARF3-R	TTAGTATATATCATAGTTTTCAGGATTCTTTTGCA	
OE-LinearARFGAP8-F	ATGGCGTCTGAAAACTTCACCGA	
OE-LinearARFGAP8-R	TCAGAGGATTCGATCTTGAAGAT	
OE-LinearSCSA1-F	ATGGCTAGACAAGCCTCTAGA	
OE-LinearSCA1-R	CTAATTCACAAGACCCCTCTCT	
RNAi-circARFGAP8-F	GTTTTTTGGCTGTAGCAGCAGCAGTACCACAAAGGTCTACAAATT	
RNAi-circARFGAP8-R	CACAGAACAGCCTAGCAGCAGGAAAATTTGTAGACCTTTGTGGTA	
RNAi-circSCSA1-F	GTTTTTTGGCTGTAGCAGCAGCAGCATGCTGGAGGTATCATTCTT	
RNAi-circSCSA1-R	CACAGAACAGCCTAGCAGCAGGAAAAGAATGATACCTCCAGCATG	
RNAicircARF3-F	GTTTTTTGGCTGTAGCAGCAGCAGCCAGTTCCTGATGTATGGGAA	
RNAicircARF3-R	CACAGAACAGCCTAGCAGCAGGAATTCCCATACATCAGGAACTGG	
phk_F	ACACGGGGGACTTTGCAAC	

Supplementary table S2

Name	Chr_Start_End_Strand	Gene
HbcircTAF12	LG02_92295427_92298349_+	LOC110646615
HbcircARF3	LG04_9262444_9263092_-	LOC110650964
HbcircTTL1	LG09_58585131_58586854_-	LOC110670358
HbcricSCSA1	LG03_62633794_62635128_-	LOC110657393
HbcircARFGAP8	LG09_17800838_17801270_-	LOC110656620
HbcircFAAH	LG16_40914365_40914884_-	LOC110644151
HbcircSMPD1	LG18_6118275_6120184_-	LOC110646088
HbcircCEMP	LG05_78561612_78562233_-	LOC110663331
HbcircABP	LG01_43254529_43256334_-	LOC110639187
HbcircARMSP	LG02_87009198_87014455_-	LOC110663402
HbcircGMI1	LG10_66804109_66807728_-	LOC110633695
HbcircSUGP1	LG15_1422229_1423458_-	LOC110643685
